# Supplementary material for: Acceptance of Human Papillomavirus (HPV) vaccine among the parents of eligible daughters (9–15 years) in Bangladesh: A nationwide study using Health Belief Model
Source: PLoS One. 2024 Nov 8;19(11):e0310779. doi: 10.1371/journal.pone.0310779 (PMC11548773; doi:10.1371/journal.pone.0310779)
Supplement: S1 Table — (DOCX) [file pone.0310779.s001.docx]

**S1 Table. Division-wise distribution of population and samples**

| **Division** | **Population*** | | **Sample** | |
| --- | --- | --- | --- | --- |
|  | **Frequency (N)** | **Percentage (%)** | **Frequency (n)** | **Percentage (%)** |
| Barishal | 9,100,102 | 5.51 | 127 | 5.9 |
| Chattogram | 33,202,326 | 20.10 | 433 | 20.13 |
| Dhaka | 44,215,107 | 26.77 | 561 | 26.08 |
| Khulna | 17,416,645 | 10.55 | 231 | 10.74 |
| Mymensingh | 12,225,498 | 7.40 | 160 | 7.44 |
| Rajshahi | 20,353,119 | 12.32 | 265 | 12.32 |
| Rangpur | 17,610,956 | 10.66 | 228 | 10.6 |
| Sylhet | 11,034,863 | 6.68 | 146 | 6.79 |

*Population statistics were taken from the latest population and housing census by the Bangladesh Bureau of Statistics (BBS)[1].

Ref: 1. Bangladesh Bureau of Statistics (BBS). Population & Housing Census 2022, Preliminary report. Minist Planning, Gov People’s Repub Bangladesh. 2022; 11.
