# Supplementary material for: Acceptance of Human Papillomavirus (HPV) vaccine among the parents of eligible daughters (9–15 years) in Bangladesh: A nationwide study using Health Belief Model
Source: PLoS One. 2024 Nov 8;19(11):e0310779. doi: 10.1371/journal.pone.0310779 (PMC11548773; doi:10.1371/journal.pone.0310779)
Supplement: S1 File — (DOCX) [file pone.0310779.s002.docx]

**APPENDIX-A**

**Informed Consent Form**

**NORTH SOUTH UNIVERSITY**

ID: ………………………………………… Date: ………………………….

Name of the Respondent:

……………………………………………………………………………………

We, a group of young researchers, are conducting research on the **“Acceptance of Human Papillomavirus (HPV) vaccine among the parents of eligible daughters (9-15 years) in Bangladesh: a nationwide study using Health Belief Model.”** Dr. Md. Delwer Hossain Hawlader, Associate Professor and Chairman, at North South University, is supervising this study. If you have any questions about this study, please contact Dr. Md. Delwer Hossain Hawlader at [mohammad.hawlader@northsouth.edu](mailto:mohammad.hawlader@northsouth.edu).

As a part of this study, your participation would be highly appreciated and would contribute a lot to this research study. You will be asked to answer several questions. Your identity will not be disclosed and will be kept confidential.

Your participation in this study will not involve any inconvenience or risks. If any question asked to you during the study poses embarrassment or discomfort, you are free to refuse to answer those questions. Your participation is voluntary. Refusal to participate or withdrawal of your consent or discontinuing participation in the study will not result in any penalty or loss of benefits. The results of this study will be presented anonymously.

North South University has reviewed and approved the procedures of this study. If you have any questions about this study, you should feel free to ask now or anytime throughout the study. If you have understood the nature of the study and have agreed to participate, please sign in the place, indicated below.

| …………………….….…..…………….. | ……….….…..………………………….. |
| --- | --- |
| Investigator’s Signature &Date | Participant’s Signature &Date |

**APPENDIX-B**

**Questionnaire**

**NORTH SOUTH UNIVERSITY**

**Study Title:** Acceptance of Human Papillomavirus (HPV) vaccine among the parents of eligible daughters (9-15 years) in Bangladesh: a nationwide study using Health Belief Model

| 0.1: ID no. |  |
| --- | --- |
| 0.2: Name of the participant |  |
| 0.3: Date of interview |  |

1. **Socio-demographic information**

| 1. You are the girl’s | - Mother - Father |
| --- | --- |
| 1. Your age (In years) | …………………….. |
| 1. Current marital status | - Living with a spouse - Living without a spouse - Divorced - Separated - Widowed - Prefer not to say |
| 1. Religion | - Muslim - Hindu - Christian - Buddhist - Others |
| 1. Place of residence | - Rural - Semiurban - Urban |
| 1. Educational Status (In completed years) | ………………………… |
| 1. Educational Status of your spouse (In completed years) | ………………………… |
| 1. Current Occupation | - Private job - Government job - Business person - Housewife - Student - Agriculture - Others (specify)……………………… |
| 1. Current occupation of your spouse | - Private job - Government job - Business person - Housewife - Student - Agriculture - Others (specify)…………………………… |
| 1. Are you a health worker? | - Yes - No |
| 1. Monthly household income (Tk) |  |
| 1. The number of household members |  |
| 1. The total number of children |  |
| 1. The number of daughters: |  |
| 1. Age of the daughter | - Age of daughter no 1: - Age of daughter no 2: - Age of daughter no 3: - Age of daughter no 4: - Age of daughter no 5: - Age of daughter no 6: - Age of daughter no 7: - Age of daughter no 8: - Age of daughter no 9: - Age of daughter no 10: |
| 1. My daughter has received other recommended childhood vaccines | - Yes all - Yes some - No - Unsure |
| 1. Is your adolescent daughter currently studying? | - Yes - No |
| 1. If Yes (1.17), what type of academic institution is it? | - Government - Private - Madrasha |
| 1. Location of academic institution of your adolescent daughter | - Rural - Semi-urban - Urban |
| 1. How important is your religion to you? | - Very important - Rather important - Neither important nor unimportant - Rather little important - Very little important |
| 1. How frequently do you have routine health check-ups? | - Never - Less than 1year interval - More than 1-2years interval - 2-5 years interval - More than 5 years interval |

1. **knowledge and source of information about HPV and HPV vaccine**

| **Questions** | **Response of the Participants** | |
| --- | --- | --- |
| 1. Have you ever heard about HPV (Human PapillomaVirus)?   (If the response is “No”, don’t ask the question 2.1.b, 2.8, 2.9) | - Yes | - No |
| 1. From where have you heard about HPV? |  |  |
| 1. Physicians/Nurses/Other health care professionals | - Yes | - No |
| 1. Training related to HPV | - Yes | - No |
| 1. Friends |  |  |
| 1. Family/Relatives | - Yes | - No |
| 1. School | - Yes | - No |
| 1. Pharmacy | - Yes | - No |
| 1. Advertisement | - Yes | - No |
| 1. Internet/social media | - Yes | - No |
| 1. Mass media (TV, Radio, Newspaper) | - Yes | - No |
| 1. Have you ever heard about the HPV vaccine? | - Yes | - No |
| 1. If yes (2.2.a), from where have you ever heard about the HPV vaccine? |  |  |
| 1. Physicians/Nurses/Other health care professionals | - Yes | - No |
| 1. Training related to HPV | - Yes | - No |
| 1. Friends | - Yes | - No |
| 1. Family/ Relatives | - Yes | - No |
| 1. School | - Yes | - No |
| 1. Pharmacy | - Yes | - No |
| 1. Advertisement | - Yes | - No |
| 1. Internet/social media | - Yes | - No |
| 1. Mass Media (TV, Radio, Newspaper) | - Yes | - No |
| 1. Have you ever heard about cervical cancer? | - Yes | - No |
| 2.3.b If yes (2.3.a), from where have you ever heard about cervical cancer? |  |  |
| 1. Physicians/Nurses/Other health care professionals | - Yes | - No |
| 1. Training related to HPV | - Yes | - No |
| 1. Friends | - Yes | - No |
| 1. Family/ Relatives | - Yes | - No |
| 1. School | - Yes | - No |
| 1. Pharmacy | - Yes | - No |
| 1. Advertisement | - Yes | - No |
| 1. Internet/social media | - Yes | - No |
| 1. Mass Media (TV, Radio, Newspaper) | - Yes | - No |
| 2.4.a Have you ever heard about cervical cancer vaccination? | - Yes | - No |
| 2.4.b If yes (2.4.a), from where have you ever heard about cervical cancer vaccination? |  |  |
| 1. Physicians/Nurses/Other health care professionals | - Yes | - No |
| 1. Training related to HPV | - Yes | - No |
| 1. Friends | - Yes | - No |
| 1. Family/ Relatives | - Yes | - No |
| 1. School | - Yes | - No |
| 1. Pharmacy | - Yes | - No |
| 1. Advertisement | - Yes | - No |
| 1. Internet/social media | - Yes | - No |
| 1. Mass Media (TV, Radio, Newspaper) | - Yes | - No |
| 1. Does cervical cancer only occur in females? | - Yes | - No |
| 1. Do you know that getting a Pap smear test helps early detection of cervical cancer? | - Yes | - No |
| 1. Have you (or your wife) ever done Pap Smear Test? | - Yes | - No |
| 1. Do you know that Human Papilloma Virus is the main cause of cervical cancer? | - Yes | - No |
| 1. Do you know how HPV is transmitted from one person to another? | - Yes | - No |
| 1. If yes (2.9.a), what is the primary transmission mode of HPV? |  |  |
| 1. Sexual Transmission | - Yes | - No |
| 1. Blood transfusion | - Yes | - No |
| 1. Use of public toilet | - Yes | - No |
| 1. During pregnancy (mother-to-child transmission) | - Yes | - No |
| 1. (Before asking this question, please explain to the participants that HPV Vaccination and Cervical Cancer Vaccination are the same)   Do you know that HPV Vaccination can reduce cervical cancer risk? | - Yes | - No |
| 1. What is the recommended age for the HPV vaccine? | - 7-9 years - 9-13 years - 13-15 years - Don’t know | |

1. **Health Belief Model**

| - 1. **Perceived Vulnerability / Susceptibility** | | | | | |
| --- | --- | --- | --- | --- | --- |
| **Questions** | **Strongly**  **Disagree** | **Disagree** | **Unsure** | **Agree** | **Strongly**  **agree** |
| 1. HPV can cause sexually transmitted disease |  |  |  |  |  |
| 1. HPV can cause condyloma/ genital warts |  |  |  |  |  |
| 1. There is a risk for young women to contract HPV |  |  |  |  |  |
| 1. HPV infection is a serious health concern |  |  |  |  |  |
| 1. I worry that my child might get HPV |  |  |  |  |  |

| - 1. **Perceived Severity** | | | | | |
| --- | --- | --- | --- | --- | --- |
| **Questions** | **Strongly**  **Disagree** | **Disagree** | **Unsure** | **Agree** | **Strongly**  **agree** |
| - 1. People with HPV might not have symptoms |  |  |  |  |  |
| - 1. HPV-associated warts could be uncomfortable or itchy |  |  |  |  |  |
| - 1. HPV-associated wart is a serious condition |  |  |  |  |  |
| - 1. HPV-associated cervical cancer is a serious condition |  |  |  |  |  |
| - 1. HPV-associated cervical cancer can occur in middle age |  |  |  |  |  |

| - 1. **Perceived Benefits** | | | | | |
| --- | --- | --- | --- | --- | --- |
| **Questions** | **Strongly**  **Disagree** | **Disagree** | **Unsure** | **Agree** | **Strongly**  **agree** |
| - 1. I trust vaccinations as it is getting better all the time because of research |  |  |  |  |  |
| - 1. The HPV vaccine is effective in preventing condyloma/ genital wart among my daughter |  |  |  |  |  |
| - 1. HPV vaccine is effective in preventing cervical cancer among my daughter |  |  |  |  |  |
| - 1. Vaccinated girls are less likely to get HPV than unvaccinated girls |  |  |  |  |  |
| - 1. HPV vaccination increases awareness of sexually transmitted diseases |  |  |  |  |  |

| - 1. **Perceived Barriers** | | | | | |
| --- | --- | --- | --- | --- | --- |
| **Questions** | **Strongly**  **Disagree** | **Disagree** | **Unsure** | **Agree** | **Strongly**  **agree** |
| - 1. I shall not vaccinate my daughter as it is painful |  |  |  |  |  |
| - 1. I shall not vaccinate my daughter as the HPV vaccine can cause an adverse effect |  |  |  |  |  |
| - 1. I shall not vaccinate my daughter as the HPV vaccine needs two injections |  |  |  |  |  |
| - 1. The HPV vaccine is so new that I want to wait a while before deciding if my daughter should get it |  |  |  |  |  |
| - 1. I shall vaccinate my daughter even if it is not free despite knowing that the HPV vaccine cost around 2500 taka |  |  |  |  |  |

| - 1. **Cues to Action** | | |
| --- | --- | --- |
| **Questions** | **Yes** | **No** |
| 1. Do you have a personal history of cancer? |  |  |
| 1. Do any of your friends or family members have a history of cancer? |  |  |
| 1. Do you have a personal history of cervical cancer? |  |  |
| 1. Do any of your friends or family members have a history of cervical cancer? |  |  |

1. **Acceptance Module**

| **Questions** | **Yes** | **No** | **Not Sure** |
| --- | --- | --- | --- |
| 4.1 Will you give your daughter the HPV vaccine? |  |  |  |
| 4.2 Will you give your daughter the HPV vaccine if the government provided it for free? |  |  |  |
