# Supplementary material for: Acceptance of Human Papillomavirus (HPV) vaccine among the parents of eligible daughters (9–15 years) in Bangladesh: A nationwide study using Health Belief Model
Source: PLoS One. 2024 Nov 8;19(11):e0310779. doi: 10.1371/journal.pone.0310779 (PMC11548773; doi:10.1371/journal.pone.0310779)
Supplement: Attachment — Submitted filename: PONE-D-23-37347_reviewer.pdf [file pone.0310779.s004.pdf]

PLOS ONE

Acceptance of Human Papillomavirus (HPV) vaccine among the parents of eligible daughters (9-15 years) in Bangladesh: a nationwide study using Health Belief Model

--Manuscript Draft--

**Manuscript Number:**	PONE-D-23-37347
**Article Type:**	Research Article
**Full Title:**	Acceptance of Human Papillomavirus (HPV) vaccine among the parents of eligible daughters (9-15 years) in Bangladesh: a nationwide study using Health Belief Model
**Short Title:**	Acceptance of Human Papillomavirus (HPV) vaccine among the parents of eligible daughters in Bangladesh
**Corresponding Author:**	Fahima Nasrin Eva<br>North South University<br>Dhaka, BANGLADESH
**Keywords:**	Human Papillomavirus, HPV Vaccine, Acceptance, Parents, Daughters, Bangladesh, Health Belief Model
**Abstract:**	Background<br>To align with the 2030 vision of the World Health Organization (WHO) to ensure 90% of girls receive the HPV vaccine before turning 15, Bangladesh has recently started the (HPV) vaccine campaign nationwide. Therefore, our study aimed to assess the level of its acceptance among parents of eligible daughters in Bangladesh.<br>Methods<br>This nationwide cross-sectional study was conducted among the self-identified parents of daughters in the age group of 9–15 years between June 28 and August 2, 2023, in a total sample size of 2,151 in all eight divisions of Bangladesh. The Health Belief Model was used to appraise respondents' beliefs concerning HPV and its vaccination. Adjusted odds ratios (AOR) with a corresponding 95% confidence interval (CI), and a p-value of <0.05 was considered statistically significant. R Studio Version 2023.09.0+463 was used for all analyses.<br>Results<br>The mean age of the study participants was 38.18 (±5.86) years. The overall acceptance rate of the HPV vaccine was 86.61%. The vaccine acceptance level was positively associated with all the domains of the HBM (p<0.001), except in the perceived barrier domain (p=0.489). After adjustment for other factors, it was found that higher age was associated with a decreased acceptance (AOR: 0.92; 95% CI: 0.89-0.95). The urban residents exhibited 42% lower odds of vaccine acceptance than rural (AOR: 0.58; 95% CI: 0.36-0.92). Similarly, participants of the middle-income group had 44% lower odds than the lower-income group (AOR: 0.56; 95% CI: 0.32-0.97).<br>Conclusion<br>Our study found a very high level of acceptance of the HPV vaccine among the parents of eligible daughters. The vaccine acceptance was positively associated with almost all the domains of the HBM. Multiple factors such as age, residence, education, income, history of medical check-ups, knowledge of cervical cancer, and cues to actions were associated with HPV vaccine acceptance.
**Order of Authors:**	Mohammad Delwer Hossain Hawlader
	Fahima Nasrin Eva
	Md. Abdullah Saeed Khan
	Tariful Islam
	Umme Kulsum Monisha
	Irin Chowdhury
	Rifat Ara
	Nur-E-Safa Meem

	Mohammad Ali Hossain
	Arpita Goutam
	Tahmina Zerin
	Nishat Alam
	Rima Nath
	Shamma Sifat
	Sayla Sultana
	Mosammat Sadeka Sultana
	Sumit Kumar Saha
	Naifa Enam Sarker
	Mohammad Hayatun Nabi
	Mohammad Lutfor Rahman
**Additional Information:**	
**Question**	**Response**
**Financial Disclosure**<br><br>Enter a financial disclosure statement that describes the sources of funding for the work included in this submission. Review the submission guidelines for detailed requirements. View published research articles from *PLOS ONE* for specific examples.<br><br>This statement is required for submission and **will appear in the published article** if the submission is accepted. Please make sure it is accurate.<br><br>**Funded studies**<br>Enter a statement with the following details:<br>• Initials of the authors who received each award<br>• Grant numbers awarded to each author<br>• The full name of each funder<br>• URL of each funder website<br>• Did the sponsors or funders play any role in the study design, data collection and analysis, decision to publish, or preparation of the manuscript?<br><br>Did you receive funding for this work?	The author(s) received no specific funding for this work.
**Competing Interests**	The authors have declared that no competing interests exist.

Powered by Editorial Manager® and ProduXion Manager® from Aries Systems Corporation

Use the instructions below to enter a competing interest statement for this submission. On behalf of all authors, disclose any competing interests that could be perceived to bias this work—acknowledging all financial support and any other relevant financial or non-financial competing interests.<br><br>This statement is required for submission and **will appear in the published article** if the submission is accepted. Please make sure it is accurate and that any funding sources listed in your Funding Information later in the submission form are also declared in your Financial Disclosure statement.<br><br>View published research articles from *PLOS ONE* for specific examples.<br><br>**NO authors have competing interests**<br><br>Enter: *The authors have declared that no competing interests exist.*<br><br>**Authors with competing interests**<br><br>Enter competing interest details beginning with this statement:<br><br>*I have read the journal's policy and the authors of this manuscript have the following competing interests: [insert competing interests here]*<br><br>* typeset	
**Ethics Statement**<br><br>Enter an ethics statement for this submission. This statement is required if the study involved:<br><br>• Human participants<br>• Human specimens or tissue<br>• Vertebrate animals or cephalopods<br>• Vertebrate embryos or tissues<br>• Field research	Approval of the research protocol: Approval was received from the Institutional Review Board (IRB)/Ethical Review Committee (ERC) of North South University.<br>Approval number: (2023/ORNSU/IRB/0507)<br>Informed Consent: Written informed consent was obtained during survey completion.

Write "N/A" if the submission does not require an ethics statement.

General guidance is provided below. Consult the submission guidelines for detailed instructions. **Make sure that all information entered here is included in the Methods section of the manuscript.**

Format for specific study types

Human Subject Research (involving human participants and/or tissue)

- Give the name of the institutional review board or ethics committee that approved the study
- Include the approval number and/or a statement indicating approval of this research
- Indicate the form of consent obtained (written/oral) or the reason that consent was not obtained (e.g. the data were analyzed anonymously)

Animal Research (involving vertebrate animals, embryos or tissues)

- Provide the name of the Institutional Animal Care and Use Committee (IACUC) or other relevant ethics board that reviewed the study protocol, and indicate whether they approved this research or granted a formal waiver of ethical approval
- Include an approval number if one was obtained
- If the study involved *non-human primates*, add *additional details* about animal welfare and steps taken to ameliorate suffering
- If anesthesia, euthanasia, or any kind of animal sacrifice is part of the study, include briefly which substances and/or methods were applied

Field Research

Include the following details if this study involves the collection of plant, animal, or other materials from a natural setting:

- Field permit number
- Name of the institution or relevant body that granted permission

Powered by Editorial Manager® and ProduXion Manager® from Aries Systems Corporation

**Data Availability**<br><br>Authors are required to make all data underlying the findings described fully available, without restriction, and from the time of publication. PLOS allows rare exceptions to address legal and ethical concerns. See the PLOS Data Policy and FAQ for detailed information.<br><br>A Data Availability Statement describing where the data can be found is required at submission. Your answers to this question constitute the Data Availability Statement and **will be published in the article**, if accepted.<br><br>**Important:** Stating 'data available on request from the author' is not sufficient. If your data are only available upon request, select 'No' for the first question and explain your exceptional situation in the text box.<br><br>Do the authors confirm that all data underlying the findings described in their manuscript are fully available without restriction?	No - some restrictions will apply
**Describe where the data may be found in full sentences. If you are copying our sample text, replace any instances of XXX with the appropriate details.**<br><br>• If the data are **held or will be held in a public repository**, include URLs, accession numbers or DOIs. If this information will only be available after acceptance, indicate this by ticking the box below. For example: *All XXX files are available from the XXX database (accession number(s) XXX, XXX.*).<br>• If the data are all contained **within the manuscript and/or Supporting Information files**, enter the following: *All relevant data are within the manuscript and its Supporting Information files.*<br>• If neither of these applies but you are able to provide **details of access elsewhere**, with or without limitations, please do so. For example:	All relevant data are within the manuscript and its Supporting Information files.

Data cannot be shared publicly because of [XXX]. Data are available from the XXX Institutional Data Access / Ethics Committee (contact via XXX) for researchers who meet the criteria for access to confidential data.<br><br>*The data underlying the results presented in the study are available from (include the name of the third party and contact information or URL).*<br>• This text is appropriate if the data are owned by a third party and authors do not have permission to share the data.<br><br>* typeset	
Additional data availability information:	

Powered by Editorial Manager® and ProduXion Manager® from Aries Systems Corporation

Manuscript
Click here to access/download;Manuscript;Manuscript_(Acceptance of HPV

Title: Acceptance of Human Papillomavirus (HPV) vaccine among the parents of eligible daughters (9-15 years) in Bangladesh: a nationwide study using Health Belief Model

Authors:

Mohammad Delwer Hossain Hawlader[1], Fahima Nasrin Eva[1,2*], Md. Abdullah Saeed Khan[2,3], Tariful Islam[1,2], Umme Kulsum Monisha[2,6], Irin Chowdhury[1,2], Rifat Ara[2,7], Nur-E-Safa Meem[1,2], Mohammad Ali Hossain[2,4], Arpita Goutam[1,2], Tahmina Zerin[1,2], Nishat Alam[1,2], Rima Nath[1,2], Shamma Sifat[1,2], Sayla Sultana[1,2], Mosammat Sadeka Sultana[1,2], Sumit Kumar Saha[1,2], Naifa Enam Sarker[1,2], Mohammad Hayatun Nabi[1], Mohammad Lutfor Rahman[5]

Affiliation:

1. Department of Public Health, North South University, Dhaka, Bangladesh
2. Public Health Promotion and Development Society (PPDS), Dhaka, Bangladesh
3. National Institute of Preventive and Social Medicine (NIPSOM), Dhaka, Bangladesh
4. Ibn Sina Medical College Hospital, Kallyanpur, Dhaka, Bangladesh
5. Institute of Statistical Research and Training (ISRT), University of Dhaka, Dhaka, Bangladesh
6. Mandy Dental College & Hospital, Dhaka, Bangladesh
7. Institute for Global Health, University College London, United Kingdom

***Corresponding author:**

E-mail: fahima.eva@northsouth.edu (FNE)

Abstract

Background

To align with the 2030 vision of the World Health Organization (WHO) to ensure 90% of girls receive the HPV vaccine before turning 15, Bangladesh has recently started the (HPV) vaccine campaign nationwide. Therefore, our study aimed to assess the level of its acceptance among parents of eligible daughters in Bangladesh.

Methods

This nationwide cross-sectional study was conducted among the self-identified parents of daughters in the age group of 9–15 years between June 28 and August 2, 2023, in a total sample size of 2,151 in all eight divisions of Bangladesh. The Health Belief Model was used to appraise respondents' beliefs concerning HPV and its vaccination. Adjusted odds ratios (AOR) with a corresponding 95% confidence interval (CI), and a p-value of <0.05 was considered statistically significant. R Studio Version 2023.09.0+463 was used for all analyses.

Results

The mean age of the study participants was 38.18 (±5.86) years. The overall acceptance rate of the HPV vaccine was 86.61%. The vaccine acceptance level was positively associated with all the domains of the HBM (p<0.001), except in the perceived barrier domain (p=0.489). After adjustment for other factors, it was found that higher age was associated with a decreased acceptance (AOR: 0.92; 95% CI: 0.89-0.95). The urban residents exhibited 42% lower odds of vaccine acceptance than rural (AOR: 0.58; 95% CI: 0.36-0.92). Similarly, participants of the middle-income group had 44% lower odds than the lower-income group (AOR: 0.56; 95% CI: 0.32-0.97).

Conclusion

Our study found a very high level of acceptance of the HPV vaccine among the parents of eligible daughters. The vaccine acceptance was positively associated with almost all the domains of the HBM. Multiple factors such as age, residence, education, income, history of medical check-ups, knowledge of cervical cancer, and cues to actions were associated with HPV vaccine acceptance.

Introduction

The global surge in HPV (Human Papillomavirus) infections is a critical concern today. HPV contributes to around 500,000 annual cancer cases, including cervical, vulvar, anal, penile, and oropharyngeal cancer(1). Ninety-nine percent of cervical cancer cases in females result from sexually transmitted HPV(2). Cervical cancer is the fourth most common cancer among women, with a global age-standardized incidence rate of 13.3/100,000 women and a mortality rate of 7.3/100,000 women(3). In Bangladesh, cervical carcinoma is the second leading malignancy in women, causing 12,000 new cases and over 6,000 deaths annually(4). HPV, specifically high-risk strains HPV-16 and 18, accounts for over 70% of cervical cancer cases worldwide(5).

The vaccination against HPV emerges as a pivotal strategy for significantly reducing cervical cancer incidence, offering both bivalent and quadrivalent options. While bivalent vaccines primarily protect against high-risk HPV varieties (HPV 16, 18), quadrivalent vaccines target both low and high-risk HPV strains (HPV 6, 11, 16, and 18)(6). Moreover, screening methods like the Pap smear, visual examination with acetic acid, and self-sampling for HPV DNA testing are effective in cervical cancer prevention(7). However, eliminating sexual risk factors and vaccination are the primary prevention methods(8). The WHO recommends vaccinating girls aged 9-14 as the primary target group, with females ≥15 as the secondary group(9).

Though vaccination is an effective way to reduce cervical cancer, there exists a distinction in vaccination coverage based on the development status of the country. In high-income countries, the coverage is almost 80%. Although data on low-and-middle-income countries and low-income countries are scarce, one study reported that in low-and-middle-income countries, the vaccination coverage is about 64%(7).

With a vision for 2030, WHO aims to ensure that 90% of girls receive the HPV vaccine before turning 15(10). The success of this ambitious goal depends largely on the broad acceptance of the vaccine, a factor closely tied to knowledge and awareness about cancer prevention measures among both the target population and their parents(6,11). However, there is a lack of comprehensive, nationwide cross-sectional studies regarding the acceptance of the HPV vaccine among the parents of adolescent girls in Bangladesh. The Government of Bangladesh, with support from UNICEF, the Vaccine Alliance (Gavi), and WHO, initiated a groundbreaking Human

Papillomavirus (HPV) vaccination campaign on October 2, 2023(12). Therefore, our nationwide study aimed to assess the level of acceptance of HPV vaccines among parents of adolescent girls in Bangladesh before the national rollout, with the ultimate goal of ensuring the program's success.

Methodology

Study design and participants

We conducted a nationwide cross-sectional study among the self-identified parents of daughters in the age group of 9–15 years in Bangladesh. Parents with foreign nationality and/or with known mental illnesses were excluded. To ensure representative sampling, we determined division-specific sample sizes using the 2022 Population & Housing Census(13), resulting in a total adjusted sample size of 2,160 participants, while considering an 80% vaccine acceptance among parents based on the existing literature and 10% non-response. We collected samples conveniently from all eight divisions (Dhaka, Mymensingh, Chattogram, Sylhet, Rajshahi, Khulna, Rangpur, and Barisal) of Bangladesh between June 28 and August 2, 2023, resulting in a total sample size of 2,151 after exclusions.

Study procedure

A team of 20 competent and trained public health graduates and students collected data from the respondents through face-to-face interviews. They were instructed to speak with as many parents as they could, regardless of their backgrounds, since a convenience sampling technique was used. To avoid linguistic hurdles, the team allocated and trained interviewers according to their locality. Participants were approached in public settings such as hospitals, schools, pharmacies, food markets, roadways, offices, and houses. Along with interacting with the general public, relatives, friends, neighbours, and colleagues of the respondents were invited for the interview. To make the questionnaire easier for the study participants to understand, we translated the difficult terms into a straightforward, native version. While answering the questionnaire, our interviewers provided explanations to participants to assist them understand certain items. Participation was entirely voluntary, and written informed consent was obtained prior to enrolment in the study.

Questionnaire

We used a semi-structured questionnaire to conduct face-to-face interviews. The questionnaire consisted of sections on 1) socio-demographic information, vaccination history of daughters, and health behaviour (21 items); 2) knowledge and information sources related to HPV and the HPV vaccine (11 items); 3) HBM constructs surrounding HPV and HPV vaccine (24 items); 4) acceptance of HPV vaccine (2 items). After a comprehensive literature review, an English draft questionnaire was developed based on previous studies(14–16). The questions were developed to cover the domains of the HBM model, which was proposed by a group of social psychologists at the US Public Health Service(17). We modified and adjusted the framing and wording of questions to reflect Bangladesh's socio-cultural and healthcare context. Following that, a Bangla translation of the questionnaire was made. Translated versions were validated by repeated revisions and agreement among the researchers. Forward-background translation by certified translators and review by relevant experts were outside the scope of the investigation due to the time-sensitive nature of the study. However, in two steps, the questionnaire's face validity was ensured. First, we shared the tool with the study supervisor and other researchers in order to obtain expert feedback on its clarity, relevance, and significance. Second, a pre-test was conducted among 28 parents of daughters aged between 9–15 years old prior to the start of the actual data collection. In order to make the questionnaire more understandable for the participants and to improve face- and construct validity, some questions were rewritten or clarified. For the pilot study, we chose participants from a wide range of socioeconomic backgrounds. In order to maintain consistency with recent literature, participant amendments were taken into account and incorporated into the survey. Following a thorough discussion, the authors finalized the questionnaire (58 items) and distributed it for the purpose of the study. Per participant, the questionnaire took about 10 to 12 minutes to complete.

Variables and measurement

Socio-demographic information, previous vaccination history of daughters, religious belief, and health profile

The survey categorized participants as either mothers (i.e., females) or fathers (i.e., males). Comprehensive personal information encompassing address, age, marital status, religion, residence, education, occupation, average monthly family income, family size, number of children, and daughters' ages were collected. Binary questions assessed the healthcare worker status and daughters' educational enrollment. Childhood vaccinations were classified as received or not. Responses indicating "yes all" or "yes some" for other recommended vaccines were grouped as "yes," whereas "no" or "not sure" responses were categorized as "others." Academic institution type (government, private, madrasa) and location (rural, semi-urban, urban) were assessed as nominal variables. Health check-up frequency was measured on an ordinal scale: "never," "<1 year," "1-2 years," "2-5 years," and ">5 years."

Knowledge and source of information about HPV and HPV vaccine

Respondents' knowledge about HPV and its vaccine and cervical cancer was assessed through eleven questions. If respondents answered affirmatively in awareness-related questions, their sources of information were sought.

HBM constructs surrounding HPV and HPV vaccine

The Health Belief Model was employed to appraise respondents' beliefs concerning HPV and its vaccination. This evaluation encompassed five aspects: perceived susceptibility (5 items), severity (5 items), benefits (5 items), barriers (5 items), and cues to action (4 items) related to HPV and its vaccine. Respondents' perceptions of susceptibility, severity, benefits, and barriers were rated on a five-point Likert scale, ranging from "Strongly Disagree" (1 point) to "Strongly Agree" (5 points). Meanwhile, cues to action were measured using a binary 'Yes'/'No' scale, with 'Yes' scored as 1 and 'No' as 0.

HPV vaccine acceptance

HPV vaccine acceptance was evaluated using two questions on vaccinating daughters and response to a government-provided free vaccination, employing a 3-point scale (‘Yes’, ‘No and ‘Not Sure’).

Statistical analysis

All data were checked for completeness, outliers, and assumption violations prior to analysis. Descriptive statistics were used to characterize the socio-demographic information of the study participants. The responses to the questions about acceptance of the HPV vaccine- 'No' and 'Not sure' were merged to produce one response- 'No' for statistical analysis. In HBM domains, answers to each individual question were first added for perceived vulnerability, perceived barrier, perceived benefit, and perceived barrier domains. This gave a total score between 5 to 25. Then, a score of ≥20 was considered a ‘positive’, and <20 was considered a ‘negative’ response for each domain. For the cues to action domain, a positive answer in one of the four questions was considered positive for the overall domain. Knowledge and beliefs about HPV and its vaccines and their association with the acceptance of the vaccine were analyzed using the Chi-square test, Fisher’s exact test, and Welch’s Two Sample t-test where appropriate. Multivariable binary logistic regression analysis was performed to identify the determinants of HPV vaccine acceptance among parents’ sociodemographic factors, health check-up frequency, and knowledge and belief regarding the HPV and HPV vaccine and cervical cancer. Adjusted odds ratios (AOR) were expressed with a corresponding 95% confidence interval (CI). A p-value of <0.05 was considered significant for statistical tests. Statistical software R Studio Version 2023.09.0+463 was used for all analyses

Ethical statement

All the procedures were carried out in accordance with the ethical guidelines of North South University's Institutional Review Board (IRB)/Ethical Review Committee (ERC) (Approval no: 2023/OR-NSU/IRB/0507). Wherever applicable, the ethical standards outlined in the 1964 Helsinki Declaration and its subsequent amendments, or comparable ethical standards, were followed(18). During the face-to-face interviews, we obtained written informed consent from all the participants involved in the study. Before providing their written consent, all the participants

were informed that their participation was entirely voluntary and that they could withdraw participation at any time. Additionally, they were also informed that all data would be presented on a group level and that only the researchers would have access to it.

Results

Table 1 represents the characteristics of the respondents. The mean age of the study participants was 38.18 (±5.86) years. Of all, 81.40% were female, and 93.86% lived with their spouse. On average, participants had 11.37 (±4.51) years of education. Most of the participants were housewives (63.41%), while only 8.51% were healthcare workers. 51.60% of the respondents lived in an urban area, and 27.98% of households had a monthly income of 35,001-50,000 BDT. Joint families (57.12%) were more common than nuclear families (42.88%). About 31.19% of respondents had done regular health check-ups. A significant portion of the population was aware of cervical cancer (80.15%). However, only 22.55% of respondents heard about Human Papillomavirus. Approximately 48.72% of participants knew about the cervical cancer vaccination, while only 22.32% knew about the HPV vaccine. About 21.57% of participants reported feeling vulnerable due to HPV infection, 19.39% felt the severity of the HPV infection and its health risk, 38.45% thought that the vaccination was beneficial, only 0.79% thought that there were barriers that would prohibit them from giving their daughters the HPV vaccine, and 41.79% of respondents had the urge to take action against cervical cancer. The overall acceptance rate of the HPV vaccine among the participants was 86.61% (**Fig 1**).

Table 1. Characteristics of the respondents (n=2,151)

**Characteristic**	**n (%)**
Age (years)	38.18 ±5.86
Sex	
Female	1,751 (81.40)
Male	400 (18.60)
Marital Status	
Living with spouse	2,019 (93.86)

Characteristic	n (%)
Living without spouse	76 (3.53)
Others	56 (2.60)
Religion	
Islam	1,784 (82.94)
Hindu	337 (15.67)
Buddhist	4 (0.19)
Christian	26 (1.21)
Residence	
Rural	783 (36.40)
Semi-urban	258 (11.99)
Urban	1,110 (51.60)
Years of Education	11.37 ±4.51
Occupation	
Job	511 (23.76)
Business	186 (8.65)
Housewife	1,364 (63.41)
Others	90 (4.18)
Health Care Worker	183 (8.51)
Monthly household income (BDT)	
<=20000	547 (25.47)
20001-35000	569 (26.49)
35001-50000	601 (27.98)
>50000	431 (20.07)
Number of family members	5.00 (4.00 – 6.00)
Family Type	
Nuclear	922 (42.88)
Joint	1,228 (57.12)
Routine Health Checkup	
Regular	671 (31.19)
Irregular	890 (41.38)

Characteristic	n (%)
Never	590 (27.43)
Heard about the Human Papillomavirus	485 (22.55)
Heard about the HPV Vaccine	480 (22.32)
Heard about Cervical Cancer	1,724 (80.15)
Heard about Cervical Cancer Vaccination	1,048 (48.72)
Perceived Vulnerability	
Negative	1,687 (78.43)
Positive	464 (21.57)
Perceived Severity	
Negative	1,734 (80.61)
Positive	417 (19.39)
Perceived Benefit	
Negative	1,324 (61.55)
Positive	827 (38.45)
Perceived Barrier	
Negative	2,134 (99.21)
Positive	17 (0.79)
Cues to Action	
Negative	1,252 (58.21)
Positive	899 (41.79)

Continuous data was expressed as Mean ±SD and Median (IQR)

Table 2 outlines participants' responses to health belief model-related questions. Regarding the Perceived Vulnerability domain, a significant portion of respondents agreed that HPV can cause sexually transmitted diseases (39.19%), can lead to condyloma/genital warts (66.20%), poses a risk for young women (37.94% agreed), represents a serious health concern (48.35%), and expressed concern about their child contracting HPV (47.28%). On the Perceived Severity domain, 24.17% of participants agreed that individuals with HPV might not exhibit symptoms, while the

majority of the participants remained neutral (68.85%). Concerning the seriousness of conditions, a significant majority agreed (55.00%) that HPV-associated cervical cancer is a serious health concern. Furthermore, a notable portion of participants (49.37%) agreed regarding the possibility of HPV-associated cervical cancer occurring in middle age. Regarding the Perceived Benefits domain, the majority of the participants (64.71%) agreed that they trust vaccinations, citing ongoing research as a reason for their trust. Regarding cervical cancer prevention, a considerable proportion agreed (54.21%) that the HPV vaccine would effectively prevent cervical cancer in their daughters. Regarding HPV transmission, 59.65% agreed vaccinated girls are less likely to contract HPV than unvaccinated girls. Moreover, 50.26% of the participants agreed that HPV vaccination increases awareness of sexually transmitted diseases. On the Perceived Barrier domain, a substantial portion of participants strongly disagreed with several barriers. Specifically, many disagreed (67.46%) with the notion that vaccination should be avoided because of potential pain. In terms of concerns related to adverse effects, a significant number disagreed (54.81%) with this barrier, suggesting that they do not view adverse effects as a significant obstacle. Similarly, participants largely disagreed (60.25%) with the statement that the vaccine's requirement of two injections was a barrier. Regarding the perception that the HPV vaccine is new and requires waiting, participants varied in their responses, with a higher proportion agreeing (36.91%) and a notable proportion disagreeing (24.97%). Finally, regarding the cost barrier, a good number of participants (32.78%) agreed that they would vaccinate their daughters despite the cost.

Table 2. Distribution of responses to health belief model-related questions (n=2,151)

**Characteristic**	**n (%)**				
	**Strongly Disagree**	**Disagree**	**Neutral**	**Agree**	**Strongly Agree**
**Perceived Vulnerability**					
HPV can cause sexually transmitted diseases.	1 (0.05)	29 (1.35)	1,175 (54.63)	843 (39.19)	103 (4.79)
HPV can cause condyloma/genital warts.	1 (0.05)	41 (1.91)	1,424 (66.20)	613 (28.50)	72 (3.35)
There is a risk for young women to contract HPV.	4 (0.19)	44 (2.05)	1,204 (55.97)	816 (37.94)	83 (3.86)
HPV infection is a serious health concern.	4 (0.19)	18 (0.84)	870 (40.45)	1,040 (48.35)	219 (10.18)

I worry that my child might get HPV.	7 (0.33)	121 (5.63)	860 (39.98)	1,017 (47.28)	146 (6.79)
**Perceived Severity**					
People with HPV might not have symptoms.	4 (0.19)	90 (4.18)	1,481 (68.85)	520 (24.17)	56 (2.60)
HPV-associated warts could be uncomfortable or itchy.	1 (0.05)	42 (1.95)	1,306 (60.72)	739 (34.36)	63 (2.93)
HPV-associated wart is a serious condition.	1 (0.05)	21 (0.98)	1,245 (57.88)	785 (36.49)	99 (4.60)
HPV-associated cervical cancer is a serious condition.	1 (0.05)	9 (0.42)	643 (29.89)	1,183 (55.00)	315 (14.64)
HPV-associated cervical cancer can occur in middle age.	2 (0.09)	23 (1.07)	932 (43.33)	1,062 (49.37)	132 (6.14)
**Perceived Benefit**					
I trust vaccinations as it is getting better all the time because of research.	3 (0.14)	13 (0.60)	480 (22.32)	1,392 (64.71)	263 (12.23)
The HPV vaccine is effective in preventing condyloma/genital warts in my daughter.	0 (0.00)	45 (2.09)	1,170 (54.39)	845 (39.28)	91 (4.23)
HPV vaccine will be effective in preventing cervical cancer in my daughter.	1 (0.05)	22 (1.02)	833 (38.73)	1,166 (54.21)	129 (6.00)
Vaccinated girls are less likely to get HPV than unvaccinated girls.	0 (0.00)	7 (0.33)	647 (30.08)	1,283 (59.65)	214 (9.95)
HPV vaccination increases awareness of sexually transmitted diseases.	0 (0.00)	31 (1.44)	864 (40.17)	1,081 (50.26)	175 (8.14)
**Perceived Barrier**					
I shall not vaccinate my daughter as it is painful.	226 (10.51)	1,451 (67.46)	392 (18.22)	79 (3.67)	3 (0.14)
I shall not vaccinate my daughter as the HPV vaccine can cause an adverse effect.	140 (6.51)	1,179 (54.81)	600 (27.89)	219 (10.18)	13 (0.60)
I shall not vaccinate my daughter as the HPV vaccine needs two injections.	183 (8.51)	1,296 (60.25)	579 (26.92)	89 (4.14)	4 (0.19)
The HPV vaccine is so new that I want to wait a while before deciding if my daughter should get it.	62 (2.88)	537 (24.97)	672 (31.24)	794 (36.91)	86 (4.00)

I shall vaccinate my daughter even if it is not free despite knowing that the HPV vaccine costs around 2500 BDT.	69 (3.21)	555 (25.80)	722 (33.57)	705 (32.78)	100 (4.65)

In response to cues to the action domain, individuals demonstrated diverse cancer-related backgrounds. While a minority disclosed their own experiences with cancer (1.53%) or cervical cancer (1.49%), a notable majority acknowledged a cancer history (35.33%) or cervical cancer history (17.29%) within their circle of friends or family **(Fig 2)**.

The association between participants' characteristics and HPV vaccine acceptance for their daughters is illustrated in **Table 3**. The average age of the parents willing to vaccinate their girls was significantly lower than those who did not accept the vaccine ($p<0.001$). Vaccine acceptance was significantly higher among mothers (i.e., female) (87.95%) than fathers (i.e., male) ($p<0.001$). The respondents living with their spouses were notably more interested in vaccinating their daughters than those living without their spouses. ($p=0.027$). Semi-urban and rural dwellers were substantially more receptive to vaccinations than others. ($p=0.018$). A notable positive association between education level and the acceptance of vaccines was found ($p<0.001$). Job holders and healthcare workers showed a higher inclination to get their daughters vaccinated compared to their other groups ($p<0.001$). The majority of the parents who expressed acceptance of the vaccine had relatively lower monthly family income ($p=0.003$) and belonged to the nuclear family ($p<0.001$). Individuals with a history of regular (93.89%) routine health checkups were more willing to vaccinate their children than others ($p<0.001$). Moreover, a considerable peak in vaccine acceptance level was observed among parents who heard about HPV (97.53%), HPV vaccine (97.71%), cervical cancer (93.33%), and cervical cancer vaccination (95.42%) compared to those who were unaware of that information ($p<0.001$). The vaccine acceptance level was positively associated with all the domains of the HBM except in the perceived barrier domain, where no association was found ($p=0.489$). Participants perceiving lesser vulnerability (97.63%) and lesser severity (97.60%) about the HPV, as well as greater benefits regarding the HPV vaccine (97.10%) and having apparent cues to actions (91.99%), demonstrated greater willingness to vaccinate their daughters.

Table 3. Association of characteristics of the respondents with HPV vaccine acceptance

	Accepts Vaccine		
**Characteristic**	**No**, (n=288)	**Yes**, (n=1,863)	**p-value**
**Age (years)**	40.70 ±6.20	37.79 ±5.71	**<0.001**[2]
**Sex**			**<0.001**[3]
Female	211 (12.05)	1,540 (87.95)	
Male	77 (19.25)	323 (80.75)	
**Marital Status**			**0.027**[3]
Living with spouse	263 (13.03)	1,756 (86.97)	
Living without spouse	18 (23.68)	58 (76.32)	
Others	7 (12.50)	49 (87.50)	
**Religion**			0.244[4]
Islam	236 (13.23)	1,548 (86.77)	
Hindu	45 (13.35)	292 (86.65)	
Buddhist	0 (0.00)	4 (100.00)	
Christian	7 (26.92)	19 (73.08)	
**Residence**			**0.018**[3]
Rural	89 (11.37)	694 (88.63)	
Semi-urban	28 (10.85)	230 (89.15)	
Urban	171 (15.41)	939 (84.59)	
**Years of Education**	10.09 ±4.06	11.57 ±4.54	**<0.001**[2]
**Occupation**			**<0.001**[3]
Job	28 (5.48)	483 (94.52)	
Business	55 (29.57)	131 (70.43)	
Housewife	191 (14.00)	1,173 (86.00)	
Others	14 (15.56)	76 (84.44)	
**Health Care Worker**	0 (0.00)	183 (100.00)	**<0.001**[3]

	Accepts Vaccine		
**Characteristic**	**No**, (n=288)	**Yes**, (n=1,863)	**p-value**
**Monthly household income (BDT)**			**0.003**[3]
<=20000	63 (11.52)	484 (88.48)	
20001-35000	63 (11.07)	506 (88.93)	
35001-50000	106 (17.64)	495 (82.36)	
>50000	55 (12.76)	376 (87.24)	
**Family Type**			**<0.001**[3]
Nuclear	89 (9.65)	833 (90.35)	
Joint	199 (16.21)	1,029 (83.79)	
**Routine Health Checkup**			**<0.001**[3]
Regular	41 (6.11)	630 (93.89)	
Irregular	160 (17.98)	730 (82.02)	
Never	87 (14.75)	503 (85.25)	
**Heard about Human Papilloma Virus**	12 (2.47)	473 (97.53)	**<0.001**[3]
**Heard about HPV Vaccine**	11 (2.29)	469 (97.71)	**<0.001**[3]
**Heard about Cervical Cancer**	115 (6.67)	1,609 (93.33)	**<0.001**[3]
**Heard about Cervical Cancer Vaccination**	48 (4.58)	1,000 (95.42)	**<0.001**[3]
**Perceived Vulnerability**			**<0.001**[3]
Negative	277 (16.42)	1,410 (83.58)	
Positive	11 (2.37)	453 (97.63)	
**Perceived Severity**			**<0.001**[3]
Negative	278 (16.03)	1,456 (83.97)	
Positive	10 (2.40)	407 (97.60)	
**Perceived Benefit**			**<0.001**[3]
Negative	264 (19.94)	1,060 (80.06)	
Positive	24 (2.90)	803 (97.10)	

	Accepts Vaccine		
**Characteristic**	**No**, (n=288)	**Yes**, (n=1,863)	**p-value**
**Perceived Barrier**			0.489[4]
Negative	285 (13.36)	1,849 (86.64)	
Positive	3 (17.65)	14 (82.35)	
**Cues to Action**			**<0.001[3]**
Negative	216 (17.25)	1,036 (82.75)	
Positive	72 (8.01)	827 (91.99)	

[1]Mean ±SD; n (%); Median (IQR)

[2]Welch Two Sample t-test

[3]Pearson's Chi-squared test

[4]Fisher's exact test

[5]Wilcoxon rank sum test

To identify the factors influencing the acceptability of the HPV vaccine among parents of young daughters, both univariate and multivariate logistic regression analysis was carried out. (**Table 4**). After adjustment for other factors, it was found that a higher age was associated with a noticeable decrease in the parents' acceptance of vaccines for their daughters. (AOR: 0.92; 95% CI: 0.89-0.95). The urban residents exhibited 42% lower odds of vaccine acceptance than those in rural areas (AOR: 0.58; 95% CI: 0.36-0.92). Similarly, participants of the middle-income group had 44% lower odds of vaccination than the lower-income group. (AOR: 0.56; 95% CI: 0.32-0.97). Moreover, those with no history of regular health checkups had 45% lesser odds of vaccine acceptance than those with a history of regular health checkups. (AOR: 0.55; 95% CI: 0.35-0.85) Contrastingly, individuals with knowledge of cervical cancer had 4.10 times higher odds than those without such knowledge. (AOR: 4.10, 95%CI: 2.89–5.85). Concerning the perceived benefits, vaccine acceptance was 3.77-fold higher odds with positive attitudes towards the benefits of the HPV vaccine (AOR: 3.77, 95%CI: 2.32–6.35). Individuals experiencing cues to actions had 1.95 times higher odds of vaccinating their girls (AOR: 1.95, 95%CI: 1.37–2.80).

Table 4. Determinants of HPV Vaccine Acceptance

	Univariate Models			Multivariate Model		
**Characteristic**	**OR**	**95% CI**	**p-value**	**OR**	**95% CI**	**p-value**
**Age (years)**	0.92	0.90 to 0.94	**<0.001**	0.92	0.89 to 0.95	**<0.001**
**Sex**						
Female	—	—		—	—	
Male	0.57	0.43 to 0.77	**<0.001**	1.05	0.53 to 2.04	0.882
**Marital Status**						
Living with spouse	—	—		—	—	
Living without spouse	0.48	0.29 to 0.85	**0.009**	0.72	0.37 to 1.46	0.349
Others	1.05	0.50 to 2.56	0.908	0.93	0.38 to 2.56	0.886
**Residence**						
Rural	—	—		—	—	
Semi-urban	1.05	0.68 to 1.68	0.821	0.72	0.42 to 1.27	0.248
Urban	0.70	0.53 to 0.92	**0.012**	0.58	0.36 to 0.92	**0.021**
**Years of Education**	1.07	1.05 to 1.10	**<0.001**	1.02	0.97 to 1.08	0.375
**Occupation**						
Job	—	—		—	—	
Business	0.14	0.08 to 0.22	**<0.001**	0.59	0.31 to 1.10	0.098
Housewife	0.36	0.23 to 0.53	**<0.001**	0.62	0.33 to 1.11	0.118
Others	0.31	0.16 to 0.64	**<0.001**	0.63	0.27 to 1.53	0.300

	Univariate Models			Multivariate Model		
**Characteristic**	**OR**	**95% CI**	**p-value**	**OR**	**95% CI**	**p-value**
**Monthly household income (BDT)**						
<=20000	—	—		—	—	
20001-35000	1.05	0.72 to 1.52	0.814	0.85	0.54 to 1.35	0.504
35001-50000	0.61	0.43 to 0.85	**0.004**	0.56	0.32 to 0.97	**0.039**
>50000	0.89	0.61 to 1.31	0.553	0.57	0.30 to 1.08	0.084
**Family Type**						
Nuclear	—	—		—	—	
Joint	0.55	0.42 to 0.72	**<0.001**	0.79	0.56 to 1.11	0.171
**Routine Health Checkup**						
Regular	—	—		—	—	
Irregular	0.30	0.20 to 0.42	**<0.001**	0.70	0.46 to 1.07	0.105
Never	0.38	0.25 to 0.55	**<0.001**	0.55	0.35 to 0.85	**0.007**
**Heard about Human Papilloma Virus**						
No	—	—		—	—	
Yes	7.83	4.55 to 14.9	**<0.001**	1.16	0.42 to 3.49	0.783
**Heard about HPV Vaccine**						
No	—	—		—	—	
Yes	8.47	4.83 to 16.6	**<0.001**	1.45	0.49 to 4.43	0.515
**Heard about Cervical Cancer**						

	Univariate Models			Multivariate Model		
**Characteristic**	**OR**	**95% CI**	**p-value**	**OR**	**95% CI**	**p-value**
No	—	—		—	—	
Yes	9.53	7.28 to 12.5	**<0.001**	4.10	2.89 to 5.85	**<0.001**
**Heard about Cervical Cancer Vaccination**						
No	—	—		—	—	
Yes	5.79	4.23 to 8.09	**<0.001**	1.32	0.86 to 2.05	0.211
**Perceived Vulnerability**						
Negative	—	—		—	—	
Positive	8.09	4.61 to 15.8	**<0.001**	1.77	0.89 to 3.87	0.125
**Perceived Severity**						
Negative	—	—		—	—	
Positive	7.77	4.32 to 15.8	**<0.001**	1.29	0.62 to 2.93	0.523
**Perceived Benefit**						
Negative	—	—		—	—	
Positive	8.33	5.55 to 13.1	**<0.001**	3.77	2.32 to 6.35	**<0.001**
**Perceived Barrier**						
Negative	—	—		—	—	
Positive	0.72	0.23 to 3.13	0.606	0.32	0.07 to 1.87	0.166
**Cues to Action**						
Negative	—	—		—	—	

	Univariate Models			Multivariate Model		
**Characteristic**	**OR**	**95% CI**	**p-value**	**OR**	**95% CI**	**p-value**
Positive	2.39	1.82 to 3.19	**<0.001**	1.95	1.37 to 2.80	**<0.001**

OR = Odds Ratio, CI = Confidence Interval

Discussion

The acceptance of the Human Papillomavirus (HPV) vaccine has emerged as a critical determinant in the global effort to combat cervical cancer, a leading cause of morbidity and mortality among women(19). In the context of Bangladesh, where cervical cancer is the second most common cancer among females and poses a significant health burden(20), understanding the factors that influence parental acceptance of the HPV vaccine is of paramount importance. This nationwide study employs the Health Belief Model as a framework to explore and elucidate the multifaceted factors that shape HPV vaccine acceptance among Bangladeshi parents.

Our study observed an impressively high acceptance rate of the HPV vaccine among parents, with an overall acceptance rate of 86.61%. This finding demonstrates a substantial willingness among Bangladeshi parents to embrace HPV vaccination as a crucial preventive measure against cervical cancer. It's important to note the variability in global acceptance of the HPV vaccine, as approval rates in different studies range from 44% to 79%(21–26). Notably, two studies conducted in Japan and Hong Kong found that less than a third of mothers expressed the intention to vaccinate their daughters (27,28). When compared to prior research in similar contexts in India and China, where parental acceptance of the HPV vaccine was approximately 71% and 83%, respectively (11,29), our study stands out with its significantly higher acceptance rate. This indicates a promising upward trend in HPV vaccine acceptance within Bangladesh.

The sociodemographic analysis reveals pivotal factors influencing HPV vaccine acceptance among Bangladeshi parents. Notably, younger parents show a greater propensity for vaccine acceptance, aligning with previous studies conducted in comparable contexts in Iran and Ethiopia(30,31). This inclination is likely attributed to their greater exposure to health-related

information and receptiveness to novel healthcare interventions. The maternal gender emerges as a significant determinant, with mothers demonstrating higher acceptance rates than fathers, which mirrors consistent trends detected in previous research conducted in various geographic areas(32,33). This variation can be attributed to the traditional caregiving roles of mothers in healthcare decision-making. However, it's worth noting that the percentage of fathers who participated in our study was 18.60%, indicating a relatively small sample size.

A significant observation in our study was that both urban residents and individuals in the middle-income group had lower vaccine acceptance rates compared to their counterparts in rural areas and lower-income groups, respectively. This aligned with research by Liu et al. (2020)(34), which revealed that individuals who were well-educated, had higher incomes, and lived in urban areas exhibited greater vaccine hesitancy. Similarly, Wagner et al. (2019)(35) and Lin et al. (2019)(36) reported similar results, indicating lower vaccine hesitancy and fewer safety concerns in low and middle-income regions compared to high-income areas of China. This underscores the complex relationship between vaccine safety perceptions, residence, and income levels, emphasizing the need to investigate these dynamics for targeted strategies to enhance vaccine acceptance within specific demographic groups.

The study revealed a strong correlation between regular health checkups and an enhanced inclination to receive vaccinations, affirming prior research by Wheldon et al. (2023) that consistently underscored the enduring importance of readily accessible healthcare in influencing vaccination behavior(37). Furthermore, a systematic review and meta-analysis conducted by Newman et al. (2018) identified a positive association between routine child preventive check-ups and parents' willingness to accept the HPV vaccine(38). These findings collectively emphasize the key role of easily reachable healthcare in shaping vaccination behavior, highlighting the necessity for policies that promote healthcare availability to boost vaccine acceptance.

Furthermore, in the analysis, it became evident that having knowledge about cervical cancer and HPV significantly motivated vaccine acceptance. This aligned with previous research by Zouheir et al. (2016)(39), which highlighted the impact of these factors as influential. A similar trend was noted in a study by Zhou et al. (2019)(40), where parental awareness was a positive factor influencing the intention for HPV vaccination among adolescents. This emphasizes the vital need

for specific awareness initiatives targeting these sociodemographic aspects to enhance the uptake of the HPV vaccine.

The application of the Health Belief Model (HBM) to understand vaccine acceptance in this context yielded several important insights. Participants generally recognized that young women were susceptible to HPV and were worried about their children getting infected with the virus, showing a sense of vulnerability to it. Nevertheless, there was a degree of uncertainty regarding the seriousness of HPV, especially concerning the presence of symptoms. This finding corresponds with earlier research by Vermandere et al. (2016) and Yarici et al. (2023)(41,42), which highlighted differing levels of comprehension regarding the health risks linked to HPV infection due to limited knowledge and awareness. The majority of participants expressed trust in vaccinations and believed the HPV vaccine would effectively prevent cervical cancer in their daughters, reinforcing the prevailing viewpoint observed in a study by Suo et al. (2020) (43), where vaccine effectiveness played a significant role in acceptance. Participants mostly disagreed with potential barriers to HPV vaccination, such as concerns about pain, side effects, and the number of shots required. Similar findings were reported in studies by Guvenc et al. (2016) and Marlow et al. (2009)(44,45), indicating that these barriers typically do not pose significant obstacles to vaccine acceptance. In fact, these barriers were generally perceived as low, while participants held higher perceptions of susceptibility to HPV, the severity of its consequences, and the benefits of vaccination. This suggests that individuals often prioritize the perceived benefits and risks associated with HPV vaccination over the potential barriers. Furthermore, the study's results underscored the substantial impact of cues to action on vaccination behavior, as individuals who responded to these prompts were nearly twice as inclined to have their daughters vaccinated. This corresponds with earlier research conducted by Wang et al. (2018)(46), delineating the crucial significance of driving influences in vaccination choices and endorsing the efficacy of interventions directed at these factors to boost vaccination rates.

The Global Vaccine Action Plan aims to save lives by ensuring equitable vaccine access worldwide(47). In Bangladesh, the battle against cervical cancer is paramount. However, the findings of this nationwide study revealed that HPV vaccine acceptance is shaped by factors like age, maternal engagement, and healthcare access. To address this, tailored awareness campaigns, educational initiatives, and enhanced healthcare services are imperative. Overcoming barriers and

implementing effective cues can significantly enhance vaccine acceptance, a crucial step in the fight against cervical cancer.

Strengths and limitations

The strengths of this study are grounded in its nationwide coverage, offering a comprehensive understanding of HPV vaccine acceptance across diverse regions of Bangladesh. Furthermore, the structured application of the Health Belief Model as a framework allows for a systematic and theory-driven analysis of the factors influencing vaccine acceptance. Additionally, the study underwent a rigorous piloting process, which contributed to the refinement of survey instruments, ensuring the clarity and consistency of data collection. Moreover, the study also considered a wide range of sociodemographic variables, adding depth to the analysis and contributing to a more nuanced understanding of the determinants of vaccine acceptance. These collective strengths bolster the robustness, representativeness, and methodological rigor of the study's findings, providing valuable insights into the landscape of HPV vaccine acceptance in Bangladesh.

The limitations of the study include its cross-sectional design, providing a brief glimpse into HPV vaccine acceptance without the ability to establish causation or observe changes over time. The use of convenience sampling may introduce biases affecting the study's overall representation. The lack of longitudinal data hinders the exploration of acceptance trends. Additionally, the study predominantly offers quantitative insights, with limited qualitative depth, to probe into the reasons behind the socio-demographic determinants of HPV vaccine acceptance. Furthermore, the study did not thoroughly investigate external influences on acceptance, such as policy shifts or external awareness campaigns, which could have influenced attitudes and behaviours. These aspects should be taken into account when interpreting the study's outcomes.

Conclusion

Our nationwide study found a very high level of acceptance of the HPV vaccine among the parents of eligible daughters. The vaccine acceptance level was positively associated with almost all the domains of the HBM. Multiple factors such as age, residence, education, income, history of medical check-ups, knowledge of cervical cancer, having a positive attitude towards the benefits, and cues to actions were associated with HPV vaccine acceptance. However, as the awareness

level is still very low, the government should take steps to initiate awareness campaigns at all levels.

Acknowledgements

The authors would like to extend their profound gratitude to all the participants in this study, who voluntarily and spontaneously contributed to our research.

References

1. Salwa M, Abdullah Al-Munim T. Ethical issues related to human papillomavirus vaccination programs: An example from Bangladesh. Vol. 19, BMC Medical Ethics. BioMed Central Ltd.; 2018.

2. Kessels SJM, Marshall HS, Watson M, Braunack-Mayer AJ, Reuzel R, Tooher RL. Factors associated with HPV vaccine uptake in teenage girls: A systematic review. Vaccine. 2012 May 21;30(24):3546–56.

3. Sung H, Ferlay J, Siegel RL, Laversanne M, Soerjomataram I, Jemal A, et al. Global Cancer Statistics 2020: GLOBOCAN Estimates of Incidence and Mortality Worldwide for 36 Cancers in 185 Countries. CA Cancer J Clin. 2021 May;71(3):209–49.

4. Banik R, Naher S, Rahman M, Gozal D. Investigating Bangladeshi Rural Women's Awareness and Knowledge of Cervical Cancer and Attitude Towards HPV Vaccination: a Community-Based Cross-Sectional Analysis. Journal of Cancer Education. 2022 Apr 1;37(2):449–60.

5. Dereje N, Ashenafi A, Abera A, Melaku E, Yirgashewa K, Yitna M, et al. Knowledge and acceptance of HPV vaccination and its associated factors among parents of daughters in Addis Ababa, Ethiopia: a community-based cross-sectional study. Infect Agent Cancer [Internet]. 2021 Dec 3 [cited 2023 Apr 10];16(1):58. Available from: https://infectagentscancer.biomedcentral.com/articles/10.1186/s13027-021-00399-8

6. Kilic A, Seven M, Guvenc G, Akyuz A, Ciftci S. Acceptance of human papillomavirus vaccine by adolescent girls and their parents in Turkey. Asian Pacific Journal of Cancer Prevention. 2012;13(9):4267–72.

7. Spayne J, Hesketh T. Estimate of global human papillomavirus vaccination coverage: Analysis of country-level indicators. Vol. 11, BMJ Open. BMJ Publishing Group; 2021.

8. Nandwani MCR. Men's knowledge of the human papillomavirus vaccine. Nurse Pract [Internet]. 2010 [cited 2023 Sep 17];35(11):32–9. Available from: https://pubmed.ncbi.nlm.nih.gov/20975448/

9. Larebo YM, Elilo LT, Abame DE, Akiso DE, Bawore SG, Anshebo AA, et al. Awareness, Acceptance, and Associated Factors of Human Papillomavirus Vaccine among Parents of Daughters in Hadiya Zone, Southern Ethiopia: A Cross-Sectional Study. Vaccines (Basel). 2022 Dec 1;10(12).

10. Garland SM, Stanley MA, Giuliano AR, Moscicki AB, Kaufmann A, Bhatla N, et al. IPVS statement on "Temporary HPV vaccine shortage: Implications globally to achieve equity." Papillomavirus

Research [Internet]. 2020 Jun 1 [cited 2023 Oct 16];9:100195. Available from: /pmc/articles/PMC7109621/

11. Madhivanan P, Li T, Srinivas V, Marlow L, Mukherjee S, Krupp K. Human papillomavirus vaccine acceptability among parents of adolescent girls: Obstacles and challenges in Mysore, India. Prev Med (Baltim). 2014 Jul 1;64:69–74.

12. Government launches nationwide human papillomavirus (HPV) vaccination campaign [Internet]. [cited 2023 Oct 12]. Available from: https://www.unicef.org/bangladesh/en/press-releases/government-launches-nationwide-human-papillomavirus-hpv-vaccination-campaign

13. Population & Housing Census: Preliminary Report (2022): Bangladesh Bureau of Statistics [Internet]. 2022 Aug [cited 2023 Oct 28]. Available from: https://bbs.portal.gov.bd/sites/default/files/files/bbs.portal.gov.bd/page/b343a8b4_956b_45ca_872f_4cf9b2f1a6e0/2023-09-27-09-50-a3672cdf61961a45347ab8660a3109b6.pdf

14. Grandahl M, Paek SC, Grisurapong S, Sherer P, Tydén T, Lundberg P. Parents' knowledge, beliefs, and acceptance of the HPV vaccination in relation to their socio-demographics and religious beliefs: A cross-sectional study in Thailand. PLoS One [Internet]. 2018 Feb 1 [cited 2023 Aug 12];13(2). Available from: https://pubmed.ncbi.nlm.nih.gov/29447271/

15. Grandahl M, Tydén T, Westerling R, Nevéus T, Rosenblad A, Hedin E, et al. To Consent or Decline HPV Vaccination: A Pilot Study at the Start of the National School-Based Vaccination Program in Sweden. Journal of School Health. 2017 Jan 1;87(1):62–70.

16. Thomas TL, Strickland O, Diclemente R, Higgins M. An opportunity for cancer prevention during preadolescence and adolescence: stopping human papillomavirus (HPV)-related cancer through HPV vaccination. J Adolesc Health [Internet]. 2013 [cited 2023 Aug 2];52(5 Suppl). Available from: https://pubmed.ncbi.nlm.nih.gov/23298993/

17. Rosenstock: The Health Belief Model: Explaining Health... - Google Scholar [Internet]. [cited 2023 Aug 12]. Available from:

https://scholar.google.com/scholar_lookup?journal=Health+Behavior+and+Health+Education&title=The+Health+Belief+Model:+Explaining+health+behavior+through+expectancies&author=I.M.+Rosenstock&publication_year=1990&

18. Declaration of Helsinki 1964 – WMA – The World Medical Association [Internet]. [cited 2023 Aug 9]. Available from: https://www.wma.net/what-we-do/medical-ethics/declaration-of-helsinki/doh-jun1964/

19. Kaur P, Mehrotra R, Rengaswamy S, Kaur T, Hariprasad R, Mehendale SM, et al. Human papillomavirus vaccine for cancer cervix prevention: Rationale & recommendations for implementation in India. Indian J Med Res [Internet]. 2017 Aug 1 [cited 2023 Sep 28];146(2):153. Available from: /pmc/articles/PMC5761024/

20. Uddin AFMK, Sumon MA, Pervin S, Sharmin F. Cervical Cancer in Bangladesh. South Asian J Cancer [Internet]. 2023 Jan 1 [cited 2023 Sep 28];12(1):36. Available from: /pmc/articles/PMC9966158/

21. Cunningham-Erves J, Forbes L, Ivankova N, Mayo-Gamble T, Kelly-Taylor K, Deakings J. Black mother's intention to vaccinate daughters against HPV: A mixed methods approach to identify

opportunities for targeted communication. Gynecol Oncol [Internet]. 2018 Jun 1 [cited 2023 Sep 26];149(3):506–12. Available from: http://www.gynecologiconcology-online.net/article/S0090825818302191/fulltext

22. Ogilvie GS, Remple VP, Marra F, McNeil SA, Naus M, Pielak KL, et al. Parental intention to have daughters receive the human papillomavirus vaccine. CMAJ [Internet]. 2007 Dec 4 [cited 2023 Sep 26];177(12):1506–12. Available from: https://www.cmaj.ca/content/177/12/1506

23. Bryer J. Black Parents' Beliefs, Attitudes, and HPV Vaccine Intentions. http://dx.doi.org/101177/1054773813487749 [Internet]. 2013 Jun 6 [cited 2023 Sep 26];23(4):369–83. Available from: https://journals.sagepub.com/doi/10.1177/1054773813487749

24. Guerry SL, De Rosa CJ, Markowitz LE, Walker S, Liddon N, Kerndt PR, et al. Human papillomavirus vaccine initiation among adolescent girls in high-risk communities. Vaccine. 2011 Mar 9;29(12):2235–41.

25. Aragaw GM, Anteneh TA, Abiy SA, Bewota MA, Aynalem GL. Parents' willingness to vaccinate their daughters with human papillomavirus vaccine and associated factors in Debretabor town, Northwest Ethiopia: A community-based cross-sectional study. Hum Vaccin Immunother [Internet]. 2023 [cited 2023 Sep 26];19(1). Available from: /pmc/articles/PMC10026865/

26. Mabeya H, Odunga J, Broeck D Vanden. Mothers of adolescent girls and Human Papilloma Virus (HPV) vaccination in Western Kenya. Pan Afr Med J [Internet]. 2021 [cited 2023 Sep 26];38(126). Available from: /pmc/articles/PMC8051220/

27. Egawa-Takata T, Ueda Y, Morimoto A, Yoshino K, Kimura T, Nishikawa N, et al. Survey of Japanese mothers of daughters eligible for human papillomavirus vaccination on attitudes about media reports of adverse events and the suspension of governmental recommendation for vaccination. Journal of Obstetrics and Gynaecology Research [Internet]. 2015 Dec 1 [cited 2023 Sep 26];41(12):1965–71. Available from: https://onlinelibrary.wiley.com/doi/full/10.1111/jog.12822

28. Wang LDL, Lam WWT, Wu J, Fielding R. Psychosocial determinants of Chinese parental HPV vaccination intention for adolescent girls: preventing cervical cancer. Psychooncology [Internet]. 2015 Oct 1 [cited 2023 Sep 26];24(10):1233–40. Available from: https://onlinelibrary.wiley.com/doi/full/10.1002/pon.3859

29. Lin Y, Su Z, Chen F, Zhao Q, Zimet GD, Alias H, et al. Chinese mothers' intention to vaccinate daughters against human papillomavirus (HPV), and their vaccine preferences: a study in Fujian Province. Hum Vaccin Immunother [Internet]. 2021 Jan 2 [cited 2023 Sep 26];17(1):304–15. Available from: https://www.tandfonline.com/doi/abs/10.1080/21645515.2020.1756152

30. Ghojazadeh M, Naghavi-Behzad M, Azar ZF, Saleh P, Ghorashi S, Pouri AA. Parental Knowledge and Attitudes about Human Papilloma Virus in Iran. Asian Pacific Journal of Cancer Prevention [Internet]. 2012 [cited 2023 Sep 26];13(12):6169–73. Available from: http://dx.doi.org/10.7314/APJCP.2012.13.12.6169

31. Mihretie GN, Liyeh TM, Ayele AD, Belay HG, Yimer TS, Miskr AD. Knowledge and willingness of parents towards child girl HPV vaccination in Debre Tabor Town, Ethiopia: a community-based cross-sectional study. Reprod Health [Internet]. 2022 Dec 1 [cited 2023 Sep 26];19(1). Available from: /pmc/articles/PMC9188100/

32. López N, Salamanca de la Cueva I, Vergés E, Suárez Vicent E, Sánchez A, López AB, et al. Factors influencing HPV knowledge and vaccine acceptability in parents of adolescent children: results from a survey-based study (KAPPAS study). Hum Vaccin Immunother [Internet]. 2022 [cited 2023 Sep 26];18(1). Available from: /pmc/articles/PMC8993091/

33. Lee Mortensen G, Adam M, Idtaleb L. Parental attitudes towards male human papillomavirus vaccination: A pan-European cross-sectional survey Infectious Disease epidemiology. BMC Public Health [Internet]. 2015 Jul 8 [cited 2023 Sep 26];15(1):1–10. Available from: https://bmcpublichealth.biomedcentral.com/articles/10.1186/s12889-015-1863-6

34. Hu D, Martin C, Dredze M, Broniatowski DA. Chinese Social Media Suggest Decreased Vaccine Acceptance in China: An Observational Study on Weibo Following the 2018 Changchun Changsheng Vaccine Incident. Vaccine [Internet]. 2020 Mar 3 [cited 2023 Sep 27];38(13):2764. Available from: /pmc/articles/PMC7211271/

35. Wagner AL, Masters NB, Domek GJ, Mathew JL, Sun X, Asturias EJ, et al. Comparisons of Vaccine Hesitancy across Five Low- and Middle-Income Countries. Vaccines (Basel) [Internet]. 2019 Dec 1 [cited 2023 Sep 27];7(4). Available from: /pmc/articles/PMC6963484/

36. Lin Y, Lin Z, He F, Hu Z, Zimet GD, Alias H, et al. Factors influencing intention to obtain the HPV vaccine and acceptability of 2-, 4- and 9-valent HPV vaccines: A study of undergraduate female health sciences students in Fujian, China. Vaccine. 2019 Oct 16;37(44):6714–23.

37. Wheldon CW, Eaton LA, Watson RJ. Predisposing, Enabling, and Need-Related Factors Associated with Human Papillomavirus Vaccination Intentions and Uptake Among Black and Hispanic Sexual and Gender Diverse Adults in the USA. J Racial Ethn Health Disparities [Internet]. 2023 Feb 1 [cited 2023 Sep 27];10(1):237–43. Available from: https://link.springer.com/article/10.1007/s40615-021-01214-1

38. Newman PA, Logie CH, Lacombe-Duncan A, Baiden P, Tepjan S, Rubincam C, et al. Parents' uptake of human papillomavirus vaccines for their children: a systematic review and meta-analysis of observational studies. BMJ Open [Internet]. 2018 Apr 1 [cited 2023 Sep 27];8(4):e019206. Available from: https://bmjopen.bmj.com/content/8/4/e019206

39. Zouheir Y, Daouam S, Hamdi S, Alaoui A, Fechtali T. Knowledge of Human Papillomavirus and Acceptability to Vaccinate in Adolescents and Young Adults of the Moroccan Population. J Pediatr Adolesc Gynecol [Internet]. 2016 Jun 1 [cited 2023 Sep 27];29(3):292–8. Available from: http://www.jpagonline.org/article/S1083318815003745/fulltext

40. Zhou M, Qu S, Zhao L, Campy KS, Wang S. Parental perceptions of human papillomavirus vaccination in central China: the moderating role of socioeconomic factors. Hum Vaccin Immunother [Internet]. 2019 Aug 3 [cited 2023 Sep 27];15(7–8):1688–96. Available from: https://www.tandfonline.com/doi/abs/10.1080/21645515.2018.1547605

41. Vermandere H, van Stam MA, Naanyu V, Michielsen K, Degomme O, Oort F. Uptake of the human papillomavirus vaccine in Kenya: Testing the health belief model through pathway modeling on cohort data. Global Health [Internet]. 2016 Nov 15 [cited 2023 Sep 28];12(1):1–13. Available from: https://globalizationandhealth.biomedcentral.com/articles/10.1186/s12992-016-0211-7

42. Yarıcı F, Mammadov B. An analysis of the knowledge of adults aged between 18 and 45 on HPV along with their attitudes and beliefs about HPV vaccine: the Cyprus case. BMC Womens Health [Internet]. 2023 Dec 1 [cited 2023 Sep 28];23(1):1–10. Available from: https://bmcwomenshealth.biomedcentral.com/articles/10.1186/s12905-023-02217-2

43. Suo T, Lu Q. Parental Acceptability of HPV Vaccination for Adolescent Daughters and Associated Factors: A Cross-Sectional Survey in Bozhou, China. Res Theory Nurs Pract [Internet]. 2020 Nov 16 [cited 2023 Sep 28];34(4):340–57. Available from: https://connect.springerpub.com/content/sgrrtnp/34/4/340

44. Guvenc G, Seven M, Akyuz A. Health Belief Model Scale for Human Papilloma Virus and its Vaccination: Adaptation and Psychometric Testing. J Pediatr Adolesc Gynecol [Internet]. 2016 Jun 1 [cited 2023 Sep 27];29(3):252–8. Available from: http://www.jpagonline.org/article/S1083318815003186/fulltext

45. Marlow LAV, Waller J, Evans REC, Wardle J. Predictors of interest in HPV vaccination: A study of British adolescents. Vaccine. 2009 Apr 21;27(18):2483–8.

46. Wang Z, Wang J, Fang Y, Gross DL, Wong MCS, Wong ELY, et al. Parental acceptability of HPV vaccination for boys and girls aged 9–13 years in China – A population-based study. Vaccine. 2018 May 3;36(19):2657–65.

47. MacDonald N, Mohsni E, Al-Mazrou Y, Kim Andrus J, Arora N, Elden S, et al. Global vaccine action plan lessons learned I: Recommendations for the next decade. Vaccine. 2020 Jul 14;38(33):5364–71.

Supporting Information

S1 File. Completed questionnaire used for data collection.

 Click here to access/download;Figure;Fig1..tif

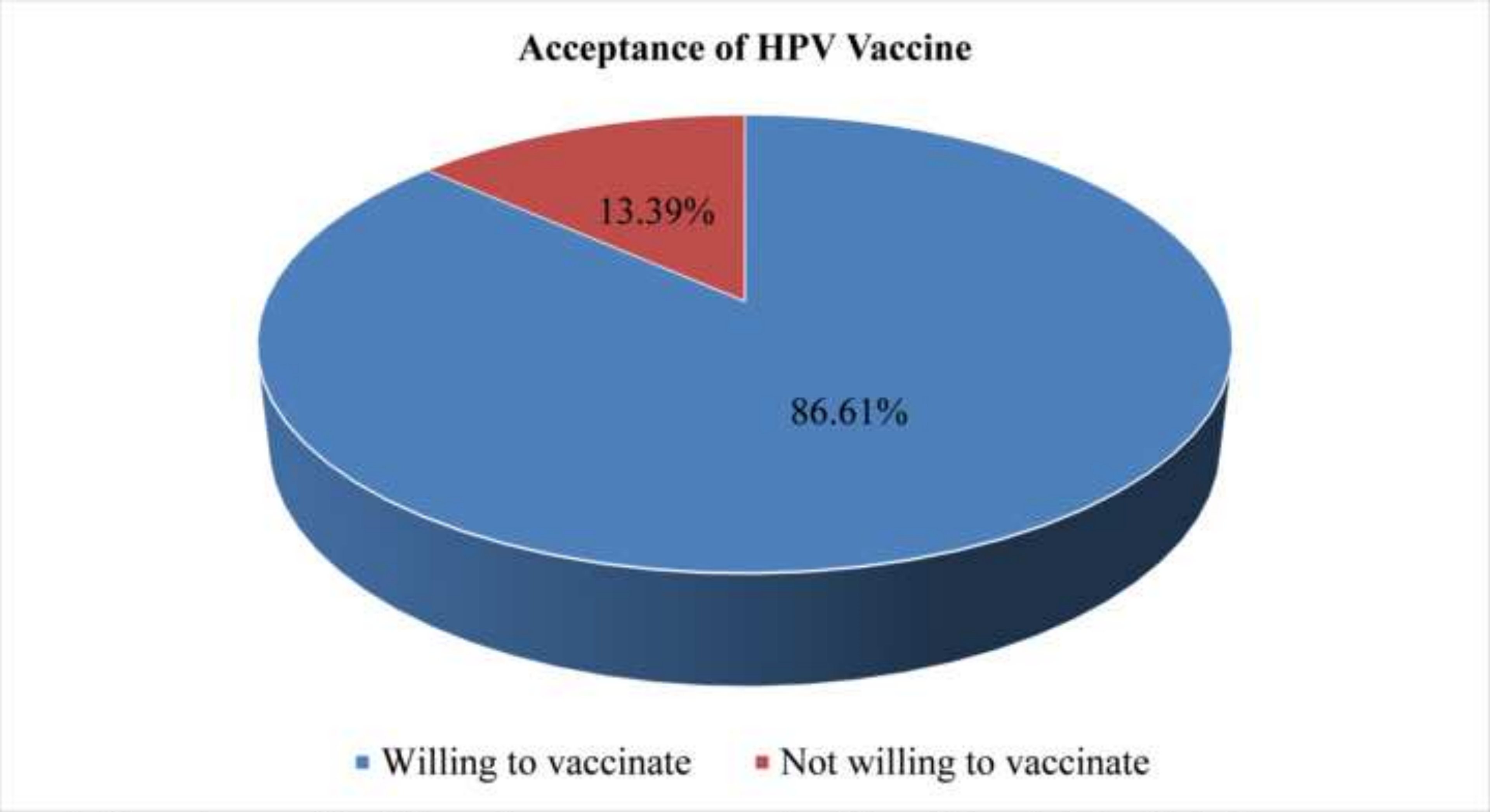

Fig 1. Acceptance of HPV vaccine among parents of daughters in Bangladesh

Figure2 Click here to access/download;Figure;Fig2.tif

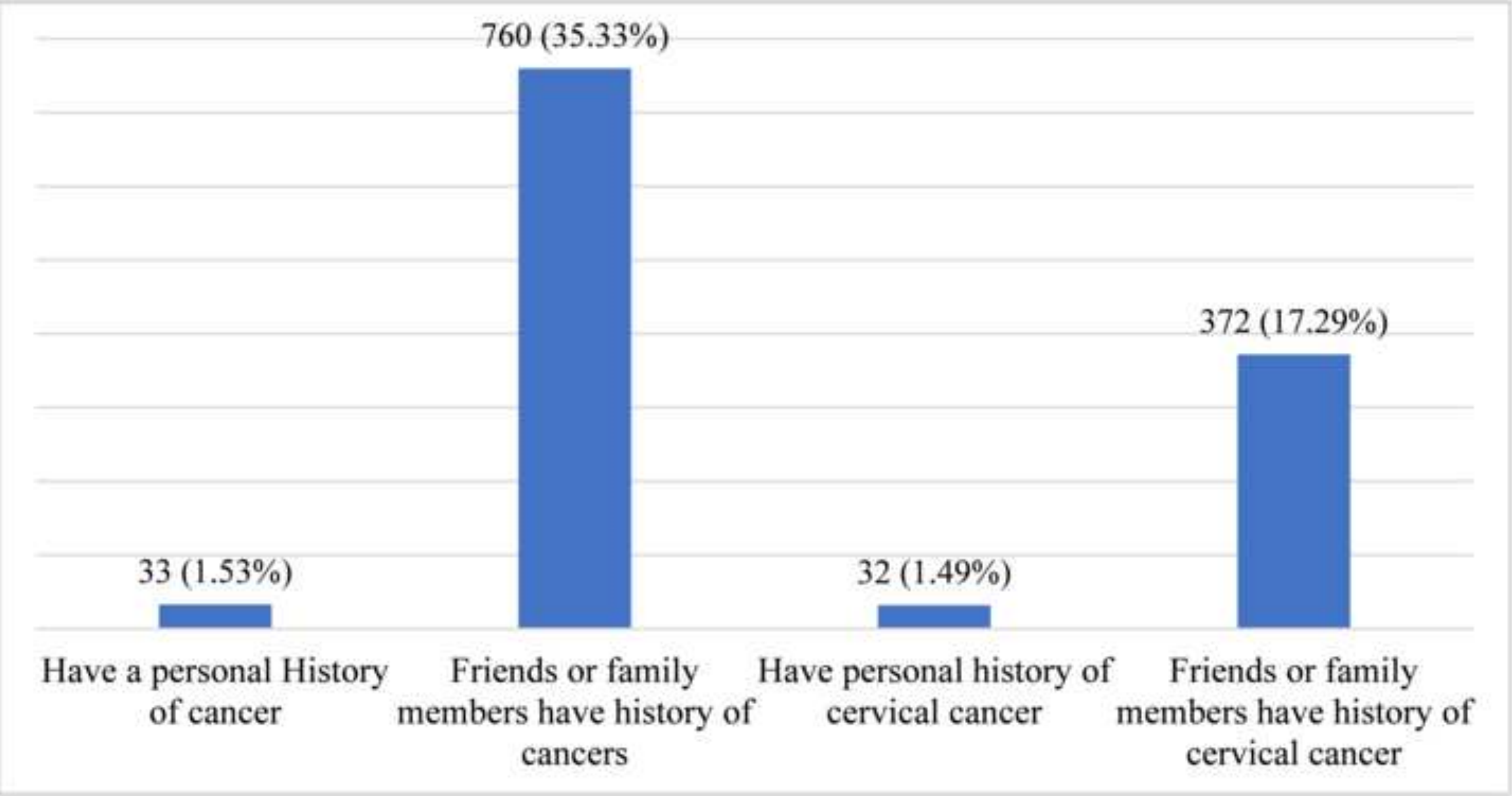

Fig 2. Cues to the action question

Full Questionnaire

Click here to access/download

Supporting Information

Final _Questionnaire.pdf
