# Supplementary material for: Acceptance of Human Papillomavirus (HPV) vaccine among the parents of eligible daughters (9–15 years) in Bangladesh: A nationwide study using Health Belief Model
Source: PLoS One. 2024 Nov 8;19(11):e0310779. doi: 10.1371/journal.pone.0310779 (PMC11548773; doi:10.1371/journal.pone.0310779)
Supplement: Attachment — Submitted filename: Response to Reviewers.docx [file pone.0310779.s005.docx]

**REBUTTAL LETTER**

**Response to Reviewer#1’s Comment:**

**Comment:** Few minor changes are required. Which being mentioned in the uploaded script. It may be considered for publication following the minor changes are made. The ethical permission part needs to be considered and Bangladesh bio-ethics society permission needs to be considered. Recommendations needs to be added in bullet points. It will provide the implication of this study.

**Response:** Thank you for your valuable feedback. Our study has received IRB approval (Approval no: 2023/OR-NSU/IRB/0507) from North South University’s Ethical Review Committee. Additionally, we have followed the ethical standards outlined in the 1964 Helsinki Declaration and its subsequent amendments wherever applicable and the STROBE guidelines to craft the manuscript (Kindly refer to the lines 193-201 in the revised manuscript). We believe this oversight adequately addresses ethical considerations.

Moreover, we have incorporated the recommendations in bullet points in the revised manuscript in the recommendation section (Please refer to lines 408-415) to highlight the implications of our study.

**Response to Reviewer#2’s Comment:**

**Comment:** Thank you Authors for coming up with this interesting manuscript. However, I have made the following review comments for better quality of the manuscript.

**1.** Under the methodology, authors should endeavor to shed more light on the content validity of the questionnaire.

Also, the total adjusted sample size was 2160 and the total sample size used after exclusion was 2151. Can authors expatiate on this disparity? Again, this is a national survey, is the sample size representative?
The pretest and pilot protocols were not referenced. Authors should kindly reference this for better understanding of the rationale behind the pretest and pilot protocol employed.

**Response:** Thank you for your comment. The questionnaire underwent a rigorous validation process and was reviewed by study supervisors and expert researchers, ensuring its appropriateness and relevance to the study objectives. Content validity was addressed through pre-testing, construct definition, item analysis, modification, pilot study and continuous review, as detailed in the questionnaire part (Please refer to lines 117-141 in the revised manuscript) under the methodology section. We believe these steps robustly address the content validity concerns of the questionnaire.

In response to the reviewer's query regarding the disparity between the total adjusted sample size (2160) and the total sample size used after exclusion (2151), we would like to clarify that the difference arises from the exclusion of participants who had incomplete data. These exclusions were necessary to ensure the integrity and validity of the study results.

In response to the concern about the representativeness of the sample size in our nationwide study, we want to highlight that we meticulously covered all eight divisions of Bangladesh and collected samples from 42 out of 64 districts. This extensive geographical coverage enhances the representativeness of our sample, ensuring a comprehensive reflection of diverse demographic and regional characteristics across the country. We have provided detailed information about the sampling strategy and coverage in the methodology section (Please refer to lines 91-116 in the revised manuscript) of the manuscript.

To address the reviewer's comment regarding the referencing of pretest and pilot protocols, we appreciate the suggestion. However, the detailed protocols for the pretest and pilot study were not published separately as part of the manuscript. Hence, direct referencing to specific pilot protocols is not feasible. To enhance clarity, we have included relevant information about the pretest and pilot study within the questionnaire section (Lines 133-138) of the methodology, providing a comprehensive understanding of the rationale and implementation. We trust this clarification meets the reviewer's expectations.

**Comment: 2.** Authors should evaluate the manuscript for grammatical errors overall. For example, Lines 295-296 do not read well. ....."Maternal gender emerges as significant determinant, with mothers demonstrating higher acceptance rate than fathers...." This should be corrected.
Overall, I will suggest a minor revision to this manuscript.

**Response:** Thank you for your valuable feedback. We have carefully reviewed and revised the manuscript for grammatical errors, including the mentioned lines. The line is now read as follows (Lines 318-320: The acceptance rate is notably influenced by sex, as females exhibit a higher acceptance rate than males….). We believe this adjustment improves the clarity and readability of the manuscript.

**Response to Reviewer#3’s Comment:**

**Comment:** I thank the Editor for inviting me to review this important manuscript. The authors have done a very good job of performing this needful study. The study has several policy implications and strengths, including large sample size, unprecedentedness, and rigor. However, I still believe the manuscript needs substantial improvements in the ways it is prepared by the authors. My comments to them are:

**Comment: 1. Lines 65 and 66**, without reference, the authors wrote: “In high-income countries, the coverage is almost 80%.”

**Response:** Thank you for bringing this to our attention. We have now appropriately referenced the statement in Lines 65-66. The relevant citations have been added to ensure accuracy and credibility. Please refer to the updated manuscript for the correct referencing (Lines 67-68).

**Comment: 2. Line 83**, the authors wrote “mental illnesses were excluded.”

**Response:** Thank you for pointing this out. We agree that the statement 'mental illnesses were excluded' can be clarified for better understanding. In the revised manuscript, we have provided a more detailed explanation of the criteria and process used for the exclusion of mental illnesses. Please refer to lines 93-94 in the revised manuscript, which is now read as follows-

“…..those who were diagnosed and were taking medication for mental health illness were excluded.”

**Comment: 3. Lines 83-84**, the authors wrote: “To ensure representative sampling, we determined division 84 specific sample sizes using the 2022 Population & Housing Census (13)”. Firstly, this statement about the study being “representative” is not objective. Second, the reference you provided opens to a page I assume to be in Bengal language. To make it easy for the authors and the reader to ascertain the study’s so-called representativeness, the authors should consider to extract the socio-demographics of Bangladeshi population from this page or any other reference and create a table showing the study’s socio-demographics on one hand and the Bangladeshi general population on the other [e.g. study’s female N 9%) vs national female N (%), study’s rural N (%) vs national rural dwellers N (%), e.t.c.]. In this way, it will be known objectively whether the study is entirely, partially, or completely not representative of the Bangladeshi population.

**Response:** Thank you very much for the suggestion. The reference you mentioned (reference no 13, now 18) opens the preliminary report of the Population and Housing Census 2022. Please note that the number ‘84’ in the sentence that you copied is the line number. It does not refer to anything else. Note also that our target was to ensure proportionate representation of the eight administrative divisions of Bangladesh. Based on your suggestion, we have included a supplementary table (S1 Table) where the population of each division with their proportion is listed alongside a number of samples taken from each division with their proportion. We extracted this data from the preliminary reports of the population and housing census available for free from the Bangladesh Bureau of Statistics. Our sample gives a complete representation of the divisions we intended to cover.

(Bangladesh Bureau of Statistics (BBS), 2022. Population & Housing Census 2022, Preliminary report. *Ministry of Planning, Government of the People’s Republic of Bangladesh*, 11. Available from: https://bbs.portal.gov.bd/sites/default/files/files/bbs.portal.gov.bd/page/b343a8b4_956b_45ca_872f_4cf9b2f1a6e0/2023-09-27-09-50-a3672cdf61961a45347ab8660a3109b6.pdf)

**Comment: 4. Lines 85-86**: without providing the reference, the authors wrote “while considering an 80% vaccine acceptance among parents based on the existing literature”

**Response:** Thank you for your observation. Referencing issues on lines 85-86 have been addressed in the revised manuscript. Please refer to line 97 in the revised manuscript to find the updated reference.

**Comment: 5. Lines 88-89:** the authors wrote “of Bangladesh between June 28 and August 2, 2023, resulting in a total sample size of 2,151 after exclusions”. Your reader may be interested in knowing the overall response rate when invitation was issued to potential participants, total responses before the exclusion, and the exclusion criteria.

**Response:** We appreciate your feedback. The methodology section of the revised manuscript now includes details on the overall response rate upon invitation, total responses before exclusions, and the exclusion criteria. Please refer to lines 101-104 in the revised manuscript, which are now read as follows-

“The overall response rate, considering those who actively participated in the survey, was approximately 90%. Of the 2160 eligible participants who agreed to participate, 2151 completed the entire questionnaire (completion rate: 99.58%); incomplete questionnaires were excluded from the analysis.”

**Comment: 6.** Also, can the authors consider attaching an English version of their study questionnaire as supplement?

**Response:** Thank you for your feedback. The English version of the study questionnaire is attached in the supplemental material section (Please refer to **S1 File**).

**Comment: 7. Lines 117-120**, why is this statement (Second, a pre-test was conducted among 28 parents of 118 daughters aged between 9–15 years old prior to the start of the actual data collection. In order to make the questionnaire more understandable for the participants and to improve face- and construct validity, some questions were rewritten or clarified.) bolder than the rest of the manuscript?

**Response:** Thank you for bringing this to our attention. The statement in lines 117-120 has been revised for better clarity. In the revised manuscript, the lines are (Line 133-135) now read as follows-

“Second, before commencing the actual data collection, a pre-test involving 28 parents of daughters aged between 9–15 years old was undertaken. To enhance participant comprehension and bolster both face- and construct validity, certain questions were revised or clarified in the questionnaire.”

**Comment: 8. Pages 4, 5, and 6** have some redundancy of information. Please, try to scale down this. You may just reference the study questionnaire you attach to the supplement.

**Response:** Thank you for your suggestion. Although, based on your suggestion, we have included the questionnaire in the supplementary table, we believe the explanatory description provided on pages 4 to 6 is not redundant. If you note carefully, you may notice that we provided explanation on how some of the responses were grouped for analyses. Also, how we operationalized knowledge in the context of this research was explained.

**Comment: 9.** How was knowledge scored?

**Response:** Thank you for the question. We explained it in the “knowledge and source of information about HPV and HPV vaccine” subsection of the method section. However, we noticed that the explanation was inadequate. Therefore, this section was modified to make it clear. The section now reads as follows-

“Knowledge was defined as the state of being aware of the term of interest in this study. Respondents' knowledge and source of information about HPV, HPV vaccine, cervical cancer, and cervical cancer-related vaccines were assessed through eight questions. First, they were asked if they had heard about HPV to investigate their awareness of HPV. If respondents answered affirmatively in the awareness-related question, their sources of information were sought. Knowledge about the HPV vaccine, cervical cancer, and cervical cancer-related vaccines was sought in a similar fashion. (lines 156 – 162).”

Hence, the knowledge-related questions had mostly binary answers and didn’t require scoring.

**Comment: 10.** Also try to disaggregate the references for each questionnaire used to assess the various domains of the study, e.g. knowledge(reference), HBM (reference)

**Response:** Thank you for your suggestions. As explained earlier, knowledge meant ‘the state of being aware (or heard) about the term.’ If one was aware, we asked them about the sources of knowledge. Hence, this section of the questionnaire was built through brainstorming and discussion among the investigators. The literature review mainly helped us formulate the HBM-related question. We have now placed the references in the exact place to make it clear in the manuscript. Please check line 123.

**Comment: 11.** On what basis did the authors classify monthly household income? One would expect the country’s minimum wage to be the reference for the income groups. Also, in no where in your manuscript did you spelt out your “BDT”

**Response:** “BDT” is the short form used globally to mean the monetary exchange note “Bangladeshi Taka”. Hence, we didn’t spell it out earlier. However, we have now spelt it in its first instance in the manuscript. Please check lines 207, 208.

**Comment: 12. Line 136**, The authors also mentioned “Academic institution type (government, private, madrasa)” as part of the study’s socio-demographic variables, which is important. However, they have not included this in the analysis without any explanation for dropping it.

**Response:** The academic institution type was the institute type of the daughters of the respondents. The question was not about the respondents (i.e., mother or father). We believe, the institute type of the respondents would have been valuable in the context of the vaccine acceptance by the respondent (not the adolescent girl). This is why this information was dropped.

**Comment: 13**. In the regression model, why have the authors not considered exploring the eight divisions of the country as potential determinants?

**Response:** Thank you for your query. We examined the possibility of including division in the model. However, as you know, the underlying calculation of logistic regression involves combinations of categories of the different variables in the multivariable regression leading to the bifurcation of samples into incrementally smaller groups. Hence, when we add division, it produces unrealistic intervals in the multivariable regression. Therefore, we decided to exclude it from the final model and discuss divisional differences in follow-up papers.

**Comment: 14**. There’s plethora of evidence supporting a parent’s/caregiver’s “level” of education with vaccination. However, I noticed the authors used “years of education” instead of “levels (primary, secondary, tertiary, university, e.t.c.”), which is more objective and informative. Why have the authors used their approach? I ask this because years of education does not necessarily mean level of education since one, for example, can decide to continue doing certificates after a primary or secondary education without progressing to a university degree.

**Response:** We choose years of education instead of level of education to incorporate the differences in institutional education among our participants. In our country, not everyone follows the same trajectory of education after secondary level. For example, some people directly go for a diploma in technical education rather than going for graduating from a university. Again, some choose the ‘Madrasa’ board, which again has two divisions (Aliya and Qaumi) instead of the national curriculum. Hence, we preferred to capture years of education instead of level of education.

**Comment: 15. Lines 287-290** in the discussion, the authors wrote: “When compared to prior research in similar contexts in India and China, where parental acceptance of the HPV vaccine was approximately 71% and 83%, respectively (11,29), our study stands out with its significantly higher acceptance rate. This indicates a promising upward trend in HPV vaccine acceptance within Bangladesh.” Why would you cite studies from India and China that reported an acceptance rate lower than that of your study and then say” This indicates a promising upward trend in HPV vaccine acceptance “within” Bangladesh. For you to say “upward trend within Bangladesh’, the reference studies have to be from Bangladesh as well.

**Response:** Thank you for pointing out the mistake. We have changed the phrasing of the sentences and given further explanation. Please check lines 309-313.

**Comment: 16. In line 295**, the authors wrote: “The maternal gender emerges…..”. What is the meaning of this phrase: and why are you now mentioning gender after you’ve explicitly mentioned in the Methods and Results that you evaluated “sex”. And why maternal? Or are you saying the women you evaluated are pregnant or what?

**Response:** Thanks again. We corrected the wording here. It now reads: “The acceptance rate is notably influenced by sex, as females exhibit a higher acceptance rate than males, which mirrors consistent trends detected in research conducted in various geographic areas”. Please check lines 318 to 320.

**Comment: 17. Lines 377 and 378 of the Conclusion**, the authors wrote: “Our nationwide study found a very high level of acceptance of the HPV vaccine among the parents of eligible daughters”, referring to the study’s acceptance rate of 86%. If we assume that, all the 86% who accepted actually have their daughters vaccinated, will this proportion meet of with the WHO target of 2030 you cited in your introduction?

**Response:** If all the participants who accept the HPV vaccine get their daughter vaccinated, it will nearly reach the expected 90% vaccination target set by WHO. However, in order to do so, we have to keep building awareness and advise people to spread the message among their peers. Thank you for the question. We believe the recommendations provided by our study, if followed, will help the authorities reach their goal of achieving the WHO-recommended target of HPV vaccination.

**Comment: 18. In the Conclusion, lines 379-381**, the authors wrote: “Multiple factors such as age, residence, education, income, history of medical check-ups, knowledge of cervical cancer, having a positive attitude towards the benefits, and cues to actions were associated with HPV vaccine acceptance.” This statement is not supported by the study findings because some of the variables mentioned were not statistically significant at the multivariate level of analysis done by the authors. Consider to revise specific to your findings.

**Response:** Thank you again for such a meticulous review. We changed the sentences. It now reads: “Multiple factors such as age, residence, family income, history of routine health check-ups, knowledge of cervical cancer, positive perception about the benefits of the vaccine, and cues to actions were associated with HPV vaccine acceptance.” Please check lines 403 to 406.

**Comment ending:** I look forward to reading the revised version of this manuscript.

**Response to Reviewer#4’s Comment:**

**Comment to authors**

Title: Acceptance of Human Papillomavirus (HPV) vaccine among the parents of eligible daughters (9-15 years) in Bangladesh: a nationwide study using Health Belief Model
Manuscript number: PONE-D-23-37347

**Abstract**

**Comment: Background** – how parent acceptance contribute to ensure 90% of girls receive the HPV vaccine before turning 15?

**Response:** Thank you for the question. In Bangladesh, the social norm is that children and adolescents do not make their own health decisions. Hence, even if the Government arranges vaccination for all eligible girls, they themselves cannot decide if they are going to be vaccinated or not. Parents are the decision-makers here. Hence, we explored the acceptance among parents.

**Comment: Method** – make it summary of method section. For instance, how was sampling conducted? How data were collected?

**Response:** Thank you for the comments. We have included these descriptions in the method section of the abstract. It now reads:

“This nationwide cross-sectional study was conducted among the self-identified parents of daughters in the age group of 9–15 years between June 28 and August 2, 2023. A total of 2,151 parents were conveniently selected from all eight divisions of Bangladesh. Data was collected through face-to-face interviews using a semi-structured questionnaire.” Please check lines 27 – 30.

**Comment: Result** – provide the finding with its upper and lower limit of the prevalence.

**Response:** Thank you. We reported the 95% confidence intervals with the proportion. Please check line 36.

**Comment: Conclusion** – what is authors’ landmark to reach at high acceptance? “The vaccine acceptance was positively associated with almost all the domains of the HBM.” What authors want to convey? If it is to indicate direction of association; all their factors are preventive.

**Response:** Thank you for your comment. We changed the statement from “very high acceptance” to “reasonably good level of acceptance”. The reason is that we considered an arbitrary landmark of nearly 90% acceptance following WHO recommendations for vaccination. However, acceptances may not necessarily translate to vaccinations. Hence, we made the change. We removed the statement “The vaccine acceptance ….of the HBM.” from the conclusion. In the sentence next to this sentence, the two domains of HBM, “perceived benefit” and “cues to action,” which showed a positive association with HPV acceptance, were stated. Hence, it was better to avoid the redundancy. Please check lines 44 to 48 to see the corrections.

**Comment: Line 45-46:** “Multiple factors such as age, residence, education, income, history of medical check-ups, knowledge of cervical cancer, and cues to actions were associated with HPV vaccine acceptance” These are crude to be used in conclusion; be specific, which classification are associated with outcome? For instance is it younger age or older? Is it having higher degree or not attending formal education?

**Response:** Thank you for the suggestion. We changed the statement accordingly. It now reads: “Multiple factors such as younger age, urban residence, monthly income in the lowest quartile, history of regular routine health check-ups, knowledge of cervical cancer, positive perception about benefits of the vaccine, and positive cues to actions were associated with HPV vaccine acceptance.” Please check lines 45 to 48. The conclusion of the main body was also changed accordingly.

**Introduction**

**Comment:** How many of the increased coverage of HPV vaccine is attributable to HPV vaccine acceptance of parent across the globe or in low and middle income countries? What is really matter? What are role of knowledge and attitude of the parents? What factors were contribute to high or low acceptance of the vaccine when you summarize previous studies? How you intend to uncover their gaps?

**Response:** Thank you for your extensive review. Based on your question we updated the background and rationale of our work. Please check lines 74 to 86.

**Method**
**Comment:** How authors maintained external validity of the finding? “We collected samples conveniently from all eight divisions” How conveniently sampled study was generalized to source?

**Response:** Thank you for the question. Our nationwide study intended to get a representation of the country's main administrative divisions. Hence, we collected data proportionately from all eight divisions, ensuring that. We added a supplementary table showing the proportionate distribution of our sample with respective divisional population distribution in Bangladesh as per the latest estimates (2022) of the Bangladesh Bureau of Statistics. However, as random sampling was not feasible for this voluntary research work, we tried to take a large sample from the population. It is well known that when random sampling is not possible, a large sample could give estimates near population means. Moreover, the participants we reached were relatively homogeneous in the context of most of the people in Bangladesh. Hence, we believe the chance of systematic bias was substantially reduced through this approach.

**Comment: Line 99-100**; how you minimize interviewer bias?

**Response:** Thank you for your question. The interviews were conducted by the research investigators, all of whom were from medical backgrounds and completed their post-graduation in public health. Moreover, these investigators were actively involved during the preparation, piloting, and finalization of the questionnaire. Also, before the final data collection, all the investigators were oriented about the questions one last time. Additionally, interviewers ensured no specific parent was given preference during data collection. They started through an arbitrary selection of parent and then continued to approach others in the school. In these ways, we tried to minimize the interviewer bias.

**Comment:** Authors should explain how they measure HPV vaccine acceptance well. The measurement for all potential variables were not described? Authors should also provide appropriate citations for measurement of outcome and explanatory variables anywhere appropriate.

**Response:** Thank you for your comment. We have included a detailed description of the variables along with their response categories in details in the ‘variables and measurement’ subsection of the methodology section. In the statistical analysis subsection, we added the following explanation of selecting multivariable logistic regression:

“Variables coming significant at bivariate analyses was included in the multivariable logistic model.” (Please check lines 188, 189).

However, please note that the selection of variables was based on a review of relevant articles, expert opinion, and discussion among investigators, in addition to their statistical significance. We also ensured that the estimates from the multivariable model do not produce unrealistic confidence ranges. Hence, as explained in response to reviewer 3, we excluded ‘division’ from this analysis.

**Comment:** How variables were selected for multivariable analysis? How model fitness were check? Was there multi-collinearity?

**Response:** Thank you for the question. The explanation for variable selection is given above. We tested model-fitness by using likelihood ratio (p<0.001), Hosmer and Lemeshow goodness of fit (GOF) test (p<0.001), and ROC curve (AUC 0.8471). The VIF test excluded multicollinearity. However, please note that, here our main interest was the coefficient estimates of each covariate, rather than the model itself. As our main aim was not the prediction of acceptance, we did not try to maximize the accuracy of the model. This is also the reason we used the ‘enter’ method during the construction of the model and included all the variables deemed necessary to investigate.

**Result**
**Comment: Line 186**: No sentence or paragraph should begin with number.

**Response:** Thank you. This was corrected. Please check line 206.

**Comment:** Table 1 and 2: what is n? Is it representing sample or frequency? Check and correct. Indentation needed for classification of variables to make easy for readers.

**Response:** In table 1 and 2 the ‘n’ represents frequency. We corrected the Indentation accordingly.

**Comment: Line 230-250**: the explanation for crude analysis are not needed. There are a lot of confounding for these association, hence you need to explain after controlling for these confounding.

**Response:** Thank you for the comment. In the lines you mentioned, we just described the direction and significance of the findings presented in Table 3. We plan to keep it for two reasons: 1. A description of the crude analysis would allow the reader to appreciate how the significance changed in multivariable analysis. 2. For readers who prefer a description over the table to understand the association’s directions.

**Comment: Can authors merge findings of table 3 and 4?**

**Response:** Thank you for the suggestion. However, we prefer to keep the tables separate for the clarity of presentation as well as the explanations described above.

**Comment: How many variables can be included in the model at once? In this study about 19 variables were run at once!**

**Response:** Industrial statistician Jim Frost in his popular book “Regression Analysis: An Intuitive Guide for Using and Interpreting Linear Models” explains that increasing covariates in multivariable models increases the accuracy of explaining the variations of dependent variables. However, this comes with a prize of increasing variance across samples (i.e., running the same model in other samples decreases the accuracy of prediction). However, the bias-variance trade-off is especially important when we are developing prediction models. In this study, we were interested in determining the degree of influence possible factors exerted on HPV vaccine acceptance. Therefore, we tried to include all the important variables in the multivariable model.

**Comment: Table 4**- what are dashes (-) represent? How about bolds?

**Response:** Thank you for the query. The dashes (-) represent reference categories, and bold represents significant p-value. We added this explanation in the table footnotes.

**Discussion**
**Comment: Line 274-280**: the discussion usually begin with purpose of the study and summary of findings.

**Response:** Thank you for your suggestion. We agree that some authors prefer putting the summary along with purpose, while others like to start directly with the main findings. We avoided putting the summary at the beginning to exclude redundancy at the conclusion. Because, the same findings were reiterated at the conclusion in a summarized form.

**Comment:** Why acceptance of the vaccine in Bangladesh were higher compared to other countries like China and Japan?

**Response:** Thank you for the question. Compared to other countries, recent nationwide vaccination for COVID-19 might have been a mover for higher acceptance of the vaccine in general, explaining the findings in our study. We included this sentence in the discussion section, too. Please check line 311 – 313.

**Comment:** Authors should explain why those in urban and middle income are lower acceptance compared to rural and low income in contradiction to previous findings.

**Response:** The higher acceptance in rural communities of Bangladesh with relatively low economic capacity could be explained by the fact that healthcare workers employed by the Ministry of Health, including health assistants and community healthcare providers, play a proactive role in increasing vaccine uptake in rural area, while the urban health care services managed by the city corporation lacks such community-centric health delivery system.

We have included these statements in line 330 to 335.

**Comment: Line 311-318:** why those who have no routine checkup have lower odds of acceptance? What really contributed to this association?

**Response:** Individuals who undergo regular health check-up are more likely be self-consciousness about their health, explaining the higher frequency of HPV vaccine acceptance. These was added in lines 341– 343.

**Comment: Line 326-348:** what are the relationship between HBM and acceptance? In your table 4, only perceived benefit and cue to action ar associated. Focus on you finding and explain why and its implications.

**Response:** Thanks a lot. Based on your suggestion we expanded the discussion section and discussed why perceived benefit and cues to action had significant association with HPV vaccine acceptance.

**Conclusion**
**Comment:** The same comments to conclusion in abstract.

**Response:** Thank you again. We changed the conclusion accordingly. Please check line no 401 – 407.


**Comment: Why Fig 1** is three dimensional? Is key is not acceptance rather willing to vaccine, do you think they are the same? Is accepting is willing to vaccinate?

**Response:** Thank you for your observations. We have updated Figure 1 to be two-dimensional and corrected the labelling to ensure clarity and avoid confusion.

**Comment:** Figure 2, what is x-y axis indicate? Label them. The figure title should fulfil its criteria.

**Response:** Thank you for your feedback. We have revised and corrected the labelling of Figure 2 to clearly indicate the x-axis and y-axis.
