# Supplementary material for: Acceptance of Human Papillomavirus (HPV) vaccine among the parents of eligible daughters (9–15 years) in Bangladesh: A nationwide study using Health Belief Model
Source: PLoS One. 2024 Nov 8;19(11):e0310779. doi: 10.1371/journal.pone.0310779 (PMC11548773; doi:10.1371/journal.pone.0310779)
Supplement: Attachment — Submitted filename: Response to reviewers.docx [file pone.0310779.s006.docx]

**REBUTTAL LETTER**

**Reviewer #2:** Authors have satisfactorily responded to my review comments. I recommend that this manuscript be accepted for publication.

**Authors Response:** Thank you for your thorough review of our manuscript.

**Reviewer #3:**

1. In Abstract Conclusion, the authors wrote “monthly income in the lowest quartile”, which is contrary to what they found from multivariable analysis. This should be middle-income since the income variable has four categories.

**Authors Response:** Thank you for your insightful observation. We have revised the wording in the abstract conclusion for clarity. The updated text now reads:

Line 46-49: “Multiple factors such as younger age, urban residence, belonging to the middle income group, history of regular routine health check-ups, knowledge of cervical cancer, positive perception about benefits of the vaccine, and positive cues to actions were associated with HPV vaccine acceptance.”

2. The Introduction is well-written and well-justified.

**Authors Response:** Thank you for your comment.

3. In the analysis section, how did the authors evaluate the fitness of their model? Consider to cite the references where applicable.

**Authors Response:** The model performance was measured using Nagelkerke’s pseudo-R-squared (0.368), Receiver Operating Characteristics Curve (Area under the curve: 0.8471), and Hosmer-Lemeshow goodness of fit test (χ2 = 2145, df = 8, p <0.001). Although the model was not a good fit, we kept the model because of its overall significance over a null model (p<0.001) and because we were interested in identifying significant determinants of vaccine acceptance rather than the predictive accuracy of the overall model.

We added this part in the methodology section. Please check lines 191 to 196.

4. I think the description of the Results section is too wordy. Doesn’t it suffice to bring out some of the most salient point and refer the rest to the appropriate figure or table.

**Authors Response:** Thank you for your feedback regarding the Results section. We understand your concern about the level of detail. However, we believe that the current description provides necessary context and clarity for the reader. The detailed narrative is essential to fully convey the complexity of our findings and their implications. We have ensured that key points are highlighted and have referred to relevant figures and tables to support and illustrate the results. We hope this approach strikes an appropriate balance between detail and readability.

5. A significant number of studies on caregiver acceptance/hesitancy of childhood vaccines has reported being male (a father) as a significant determinant. In view of this, can your low number of male participants also be a limitation? Best

**Authors Response:** Thank you for the nice suggestion. We added this as a limitation in line numbers 404 – 405.

**Reviewer #5**: It's good to see that the author has addressed quite a number of comments; however, there are a few lapses that need to be addressed:

1. Line 178 - "Perceived barrier" was repeated. Kindly address.

**Authors Response:** Thank you for your observation. We have addressed this comment in the revised manuscript. Please refer to lines 179-181 for the updated content.

2. Line 191- You mentioned that R studio was used for data analysis. Please note that R studio is an integrated development environment (IDE) for the R programming language. It would be more accurate to mention that R Studio was used as an interface for data analysis, while R (the programming language) was used for statistical computations.

**Authors Response:** Thank you for your comment. We have revised the wording for clarity and accuracy. The updated line (Line 199-201) now reads:

"R Studio (Version 2023.09.0+463) was used as an interface for data analysis, while R (the programming language) was used for statistical computations."

3. Throughout the data analysis section, there was no justification provided for the merging of responses for the HPV vaccine acceptance analysis. Therefore, please state the rationale behind merging the "No" and "Not sure" responses for the HPV vaccine acceptance analysis. This clarification assists readers in understanding the methodology and ensures transparency in the research process.

**Authors Response:** Thank you again for the detailed comment. We added clarification in the statistical analysis subsection of the methods section. Please check lines 189– 191.

4. Lastly, consider including recommendations for future research. Providing suggestions for future research based on the study's findings, such as exploring interventions to address specific barriers to vaccine acceptance or conducting longitudinal studies to track acceptance trends, could be valuable.

**Authors Response:** Thank you for your valuable suggestion. We have now incorporated a recommendations section for future research based on our study's findings. Please refer to lines 420-429 in the revised manuscript for these updates.
